# Deep sequencing and *in silico* analyses identify MYB-regulated gene networks and signaling pathways in pancreatic cancer

**DOI:** 10.1038/srep28446

**Published:** 2016-06-29

**Authors:** Shafquat Azim, Haseeb Zubair, Sanjeev K. Srivastava, Arun Bhardwaj, Asif Zubair, Aamir Ahmad, Seema Singh, Moh’d. Khushman, Ajay P. Singh

**Affiliations:** 1 Department of Oncologic Sciences, Mitchell Cancer Institute, University of South Alabama, Mobile, AL 36604, USA; 2 Molecular and Computational Biology, School of Biological Sciences, Dornsife College of Letters, Arts and Sciences, University of Southern California, Los Angeles, CA 90089, USA; 3 Department of Biochemistry and Molecular Biology, College of Medicine, University of South Alabama, Mobile, AL 36688, USA; 4 Department of Interdisciplinary Clinical Oncology, Mitchell Cancer Institute, University of South Alabama, Mobile, AL 36604, USA

## Abstract

We have recently demonstrated that the transcription factor MYB can modulate several cancer-associated phenotypes in pancreatic cancer. In order to understand the molecular basis of these MYB-associated changes, we conducted deep-sequencing of transcriptome of MYB-overexpressing and -silenced pancreatic cancer cells, followed by *in silico* pathway analysis. We identified significant modulation of 774 genes upon MYB-silencing (p < 0.05) that were assigned to 25 gene networks by *in silico* analysis. Further analyses placed genes in our RNA sequencing-generated dataset to several canonical signalling pathways, such as cell-cycle control, DNA-damage and -repair responses, p53 and HIF1α. Importantly, we observed downregulation of the pancreatic adenocarcinoma signaling pathway in MYB-silenced pancreatic cancer cells exhibiting suppression of EGFR and NF-κB. Decreased expression of *EGFR* and *RELA* was validated by both qPCR and immunoblotting and they were both shown to be under direct transcriptional control of MYB. These observations were further confirmed in a converse approach wherein MYB was overexpressed ectopically in a MYB-null pancreatic cancer cell line. Our findings thus suggest that MYB potentially regulates growth and genomic stability of pancreatic cancer cells via targeting complex gene networks and signaling pathways. Further in-depth functional studies are warranted to fully understand MYB signaling in pancreatic cancer.

Pancreatic cancer is expected to be the third major cause of cancer-related deaths in 2016 in the US. It is estimated that 53,070 individuals will be afflicted while 41,780 patients will succumb to the disease over the course of this year^1^. Despite the increase in survival for most cancers, the 5-year survival for pancreatic cancer patients has remained dismal and is ~8%^1^. Although surgery is presently the only curative treatment, a significant number of pancreatic cancers are unresectable at the time of initial diagnosis. Even for resectable pancreatic cancers, therapeutic strategies based on initial resection are less promising in alleviating the survival of patients as 80% pancreatic cancer patients suffer relapse after resection^2^.

Pancreatic cancer tumorigenesis is driven by genetic alterations that involve somatic mutations and gene rearrangements^3^. In 1997, Wallrapp and co-workers identified MYB, a proto-oncogene to be amplified in PC^4^. MYB is the cellular counterpart of v-MYB oncogene carried by chicken leukemia virus. *MYB* gene has been found to be amplified in cancers and its aberrant expression is implicated in several types of malignancies including leukemias, pancreatic, prostate, colorectal, breast, head and neck cancer and salivary gland tumor^5^,^6^,^7^,^8^,^9^,^10^,^11^. This gene encodes a transcription factor that binds to the conserved 5′-YAAC[GT]G-3′ sequences and regulates cell proliferation, survival and differentiation^5^. Recently, we established the role of MYB as a novel regulator of pancreatic tumor growth and metastasis as it modulated cancer associated phenotypes such as growth, tumorigenicity, cell cycle, migration and invasion^6^.

With the advent of next generation sequencing (NGS), high resolution genomic and transcriptomic information can be retrieved through the whole genome sequencing (WGS) and RNA sequencing (RNA-seq)^12^. The transcriptome profiling is rapidly replacing the hybridization-based microarrays as it provides an unbiased, extensive and precise measurement of levels of transcripts and their isoforms^13^. Differentially expressed genes in two or more conditions can be identified through RNA-seq and the biological significance of the transcriptomic alterations can be analysed through a number of bioinformatics tools. Ingenuity Pathway Analysis (IPA) provides one such user-friendly interface that can translate the changes in gene expression to that of altered networks and pathways^14^.

In this study, we analyzed the differential expression of genes in MYB-silenced MiaPaCa cells, relative to the MYB-expressing parental cells. The genes modulated upon MYB-silencing were annotated through comparative analysis and the biological significance of the altered transcriptome was interpreted *via* IPA. EGFR and RELA were observed to be down-regulated upon MYB-silencing and were confirmed to be direct transcriptional targets of MYB. Moreover, the MYB-induced changes in gene expression were also verified by ectopic expression of MYB in BxPC3 cell line, further strengthening the role of MYB in pancreatic cancer. Analyses of the dataset also suggested other novel functions of MYB in pancreatic cancer that warrant in-depth investigation to comprehend their functional relevance and are subject of ongoing research.

## Results

### Identification and validation of differentially-expressed genes in MYB-silenced pancreatic cancer cells

**Figure 1:**
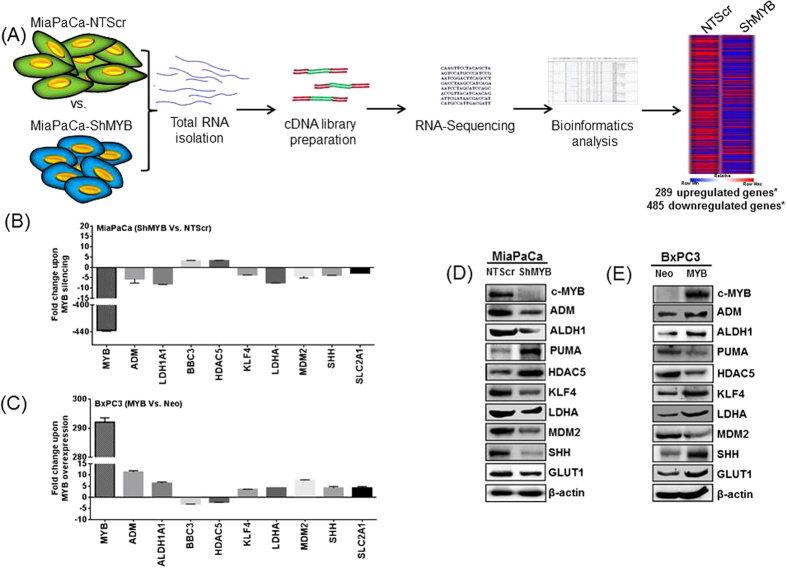
RNA sequencing and validation of select genes. (**A**) Total RNA was isolated from control (MiaPaCa-NTScr) and MYB-silenced (MiaPaCa-ShMYB) MiaPaCa cells, cDNA synthesized and further subjected to sequencing using Illumina HiSeq 2500 platform. Bioinformatics analysis revealed 774 differentially expressed genes (*p value ≤ 0.05, fold change ≥± 1.5) in the sample set. (**B**) The expression of a few select genes from the resulting MiaPaCa (shMYB Vs. NTScr) RNA-Seq data was also validated using qPCR. In a converse approach, we also compared the (**C**) relative expression of these genes in ectopic MYB-expressing (BxPC3-MYB) vs. control BxPC3 (BxPC3-Neo) cell lines. (**D,E**) The expression at protein level of these genes was also analyzed by immunoblotting. PUMA and GLUT1 represent the proteins encoded by *BBC3* and *SLC2A1* genes, respectively.

To identify the transcriptomic alterations governed by MYB in PC, we combined the traditional strategy of gene manipulation with high throughput sequencing followed by bioinformatics analysis as depicted in Fig. 1A. RNA-sequencing analysis revealed large number of genes altered upon MYB-silencing in MiaPaCa (MiaPaCa-shMYB) PC cells as compared to the control (MiaPaCa-NT-Scr) cells. The full dataset is available at the National Centre for Biotechnology Information Gene Expression Omnibus database (www.ncbi.nlm.nih.gov/geo/; accession number: GSE61290). A total of 774 differentially-expressed genes were identified by using a fold-change ≥±1.5 and p value ≤ 0.05, of which 485 were down-regulated and 289 were up-regulated in MiaPaCa-shMYB compared to MiaPaCa-NT-Scr cells. The complete list of genes, their experimental fold change, P value and annotations, are provided in Supplementary Table 1. To validate the RNA-seq data, expression of some selected genes; *c-MYB, ADM, ALDH1, BBC3* (PUMA)*, HDAC5, KLF4, LDHA, MDM2, SHH, SLC2A1* (GLUT1); relevant to PC pathobiology were examined by quantitative reverse transcription PCR (qPCR) using gene specific primer set. The change in the expression profile of these genes was consistent with the RNA-seq data (Fig. 1B). Furthermore, to ascertain the role of MYB in modulating the expression of these genes in PC, in a converse approach wherein we overexpressed MYB in low MYB expressing BxPC3 cells, the expression profile observed was *vice versa* to MYB-knockdown in MiaPaCa PC cells (Fig. 1C). The expression of these genes at protein level was also examined in high (MiaPaCa-NT-Scr and BxPC3-MYB) and low (MiaPaCa-shMYB and BxPC3-Neo) MYB-expressing PC cells by immunoblotting. The changes at the protein level were in accordance with the changes observed at the transcript level for the analyzed genes (Fig. 1D,E).

### Identification of MYB-regulated gene networks in pancreatic cancer

**Figure 2:**
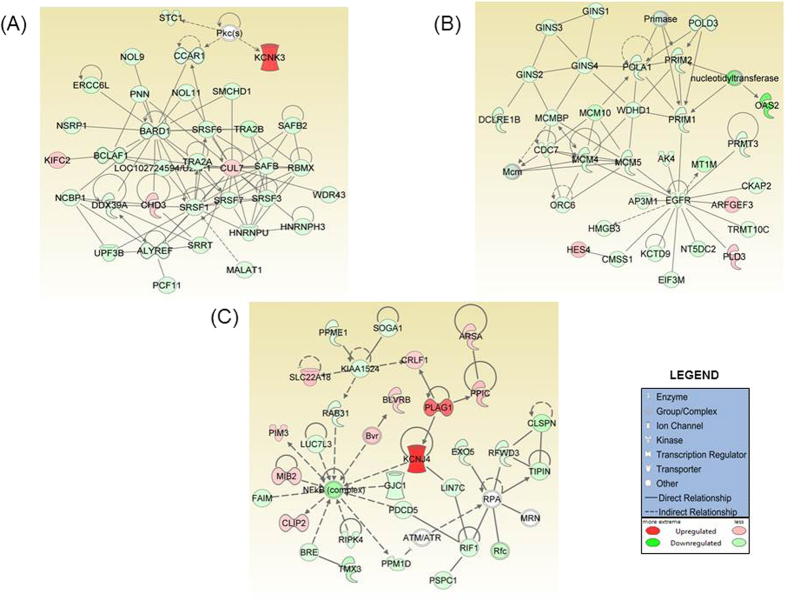
MYB-regulated gene networks predicted using Ingenuity Pathway analysis. The top three networks enriched for genes with statistically significant interconnection between the molecules present in our dataset have been presented. The highest scoring network was: (**A**) Network 1: the connected genes correlated to RNA post-transcriptional modification, molecular transport and RNA trafficking (score = 54, focus molecules = 34) functions followed by (**B**) Network 2: genes are mainly involved in cellular assembly and organization (score = 49, focus molecules = 32) with *EGFR* occupying a central node, and (**C**) Network 3: genes engaged in cell cycle, DNA replication, recombination and repair (score = 41, focus molecules = 29). The NF-κB complex is one of the central node downregulated in this network. The legend explaining the edge type and node shape are given in the bottom right panel. Direct regulatory relationships are represented by solid lines and indirect regulatory networks by dashed lines as depicted in the legend.

Next, we performed the Ingenuity Pathways Analysis (IPA) to interpret our dataset in the context of MYB-regulated gene networks in PC cells. IPA transforms the list of genes into a set of networks built on the records maintained in their database which are largely based on published reports^15^,^16^. Each network is generated algorithmically based on their connectivity and assigned a score. IPA analysis revealed 25 different gene networks upon MYB-silencing in PC cells (Supplementary Table 2). Amongst them, the top three modulated networks are presented in Fig. 2. Network 1 was predicted with a score of 54 and comprised of 34 focus molecules (Fig. 2A). In this network, thirty genes were found to be down-regulated, including several splicing factors such as *SRSF1*, *SRSF5, SRSF7* and *SRSF10,* while four genes (*KIFC2*, *CHD3*, *CUL7* and *KCNK3)* were upregulated. As revealed by the IPA software, the top function associated with this network was “RNA Post-Transcriptional Modification, Molecular Transport and RNA Trafficking”. Network 2 (Score - 49, focus molecules - 32; Fig. 2B), consisted of three upregulated (*ARFGEF3, HES4, PLD3*) and twenty-nine downregulated genes including *EGFR*, GINS complex, MCM complexes and primases, suggesting an overall downregulation of this network. The top function of the genes in this network is “Cellular Assembly and Organization”. Network 3 (Score - 41, focus molecules - 41; Fig. 2C) consisted of eleven up-regulated and eighteen down-regulated genes. NF-κB complex, RPA and *KIAA1524* occupied the central nodes of this network and were all down-regulated. As determined by IPA software, these molecules are mainly involved in “Cell cycle, DNA Replication, recombination and Repair”.

### MYB-modulated signaling pathways in pancreatic cancer

**Figure 3:**
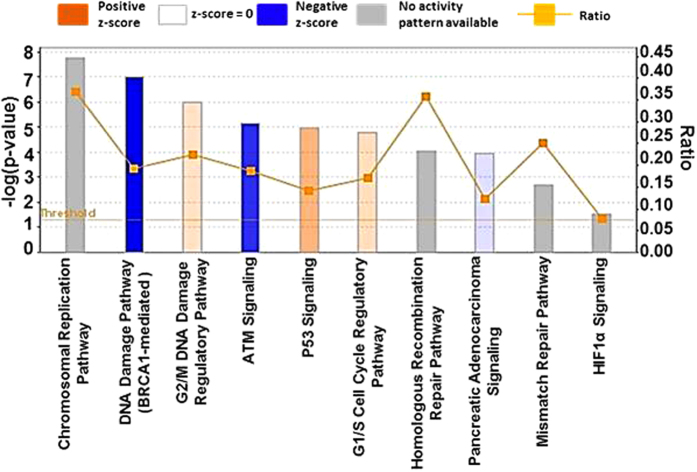
Identification of MYB-regulated canonical pathways based on the Ingenuity Knowledge Base. Ten PC-related canonical pathways identified have been illustrated. Pathways identified are represented on the *x-*axis. The left *y*-axis corresponds to the –log of the *P*-value (Fisher’s exact test) and the right *y-*axis represents the ratio (orange points) of the number of genes in a given pathway that meet cutoff criteria, divided by the total number of genes that map to that pathway.

**Figure 4:**
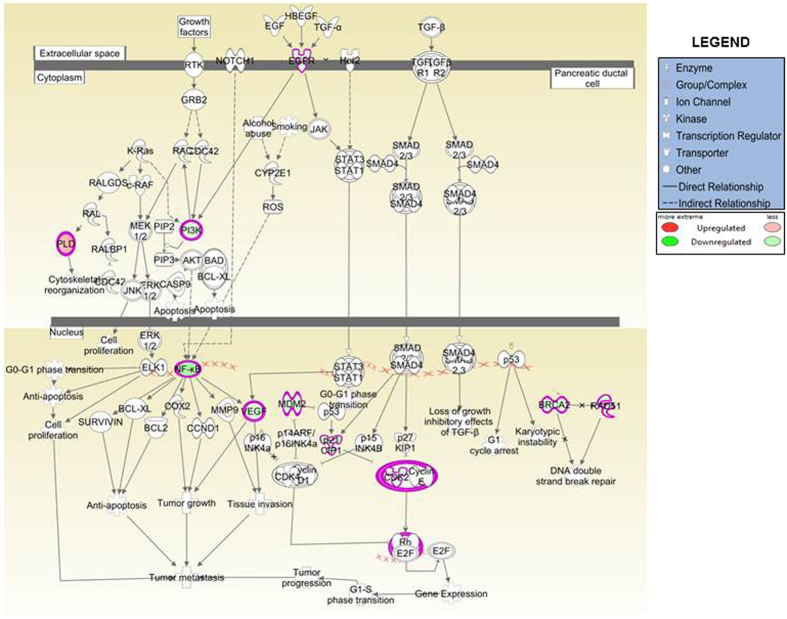
The pancreatic adenocarcinoma pathway. MYB silencing in pancreatic cancer cells led to downregulation of pancreatic adenocarcinoma pathway in our IPA analysis (*Z* score = −0.333). The schematic depicts different signaling components of this pathway and highlights the down-regulated genes and associated signaling complexes in ‘green’, while the solitary up-regulated gene, *PLD*, is presented in ‘red’.

In order to identify the signaling pathways catalogued in the IPA library that are of significance to our dataset, “canonical pathway” analysis of the IPA was employed. Ten enriched canonical signaling pathways upon MYB-silencing are represented in Fig. 3. An upregulation of pathways associated with cell cycle checkpoint regulation including “G2/M DNA Damage Regulatory Pathway” (z score: 0.302), “G1/S Cell Cycle Regulatory Pathway” (z score: 0.333), along with the upregulation of “p53 signaling” (z score: 0.632) was observed. These pathways are involved in induction of cell cycle arrest and apoptosis, and up-regulation of these pathways in MYB-silenced cells is supportive of the de-repression of cellular regulatory mechanisms in the absence of MYB. Surprisingly, “BRCA1-mediated DNA Damage Signaling” and “ATM signaling” (z score −2.53 and −1.897, respectively) were observed to be down-regulated. These signaling pathways are mostly reported to be inversely correlated with the expression of oncogenes, and further investigations are required to interpret the significance of these signaling pathways in PC, particularly in the context of MYB signaling. Further, as expected, the inhibition of “Pancreatic Adenocarcinoma Signaling” with a z-score of −0.333 was also revealed by the IPA analysis. Supplementary Table 3 lists the enriched pathways, genes involved, ratio of the genes present in our dataset to that present in IPA database, z-score and p-values. However, while changes in expression of genes involved in “Chromosomal Replication Pathway, Homologous Recombination Repair Pathway, Mismatch Repair Pathway” and “HIF-1α Signaling” was observed, IPA did not predict any activity pattern for these networks and their activation state could not be inferred. Together, this data suggests the power of IPA analysis for the quick identification of perturbed signaling pathways, and also validates the role of MYB in the regulation of several canonical pathways that are of significance to PC pathogenesis. Having observed down-regulation of pancreatic adenocarcinoma signaling pathway upon MYB-silencing, we next focused on the genes of this pathway that were present in our dataset. We observed that in our dataset, a number of factors representing pancreatic adenocarcinoma signaling pathways were down-regulated. These included EGFR, NF-κB, VEGF, PI3K, MDM2 and CDK2 (Fig. 4).

### Characterization of EGFR and RELA as direct transcriptional targets of MYB

**Figure 5:**
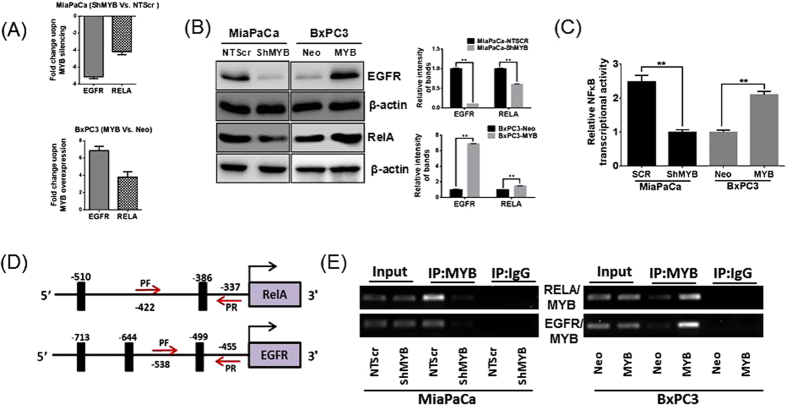
MYB regulates expression of EGFR and RELA via direct binding to their promoter regions. Expression of EGFR and RELA was analyzed by (**A**) qPCR and (**B**) immunobloting in MYB silenced MiaPaCa (-ShMYB) and MYB-overexpressing BxPC3 (-MYB) cells and their respective controls, MiaPaCa-NTScr and BxPC3-Neo. Densitometric analyses of the band intensities are represented as bar-graphs on the right (**C**) Sub-confluent MiaPaCa (-NTScr and -ShMYB) and BxPC3 (-Neo and –MYB) cell lines were co-transfected with RELA NF-κB responsive luciferase reporter and TK-Renilla luciferase (control) plasmids. After 48 hours, the cells were harvested and luciferase activity was measured. MYB-silencing in MiaPaCa cells decreased the NF-κB activity, while an enhancement in the activity of NF-κB was observed in BxPC3-MYB cells. (**D**) Putative MYB binding sites (thick black bars) in EGFR and RELA promoter region and their distance from the first codon are depicted. Arrows identify complementary sites for forward and reverse primers in the flanking region of MYB binding site(s). (**E**) Anti-MYB antibody or normal IgG antibody were used to perform ChIP analysis. Genomic fragment-associated with immunoprecipitated DNA was isolated and amplified using specific qPCR primers flanking MYB-binding site within the EGFR and RELA promoter region **p value < 0.05.

EGFR and NF-κB-p65 have been reported to be of significance to PC pathogenesis and our RNA-seq data reported a 2.214 and 4.638 fold decrease, respectively, in transcript levels of these genes. Therefore, we subsequently explored the mechanism through which the inhibition of both EGFR and NF-κB (RELA) was attained in MYB-silenced cells. We first measured the mRNA expression of *EGFR* and *RELA* in MiaPaCa-shMYB and BxPC3-MYB cells, relative to their respective controls. The qRT-PCR data showed a 7.14 and 4.02 fold decrease in transcript levels of EGFR and RELA, respectively, upon MYB silencing in MiaPaCa cells (Fig. 5A, top panel), which is consistent with their decreased expression as obtained in RNA-seq. Conversely, the mRNA expression of these genes was enhanced by forced expression of MYB in BxPC3 cells (Fig. 5A, bottom panel) with *EGFR* and *RELA* reporting a 6.54 and 3.74 fold increase in transcript levels, respectively. These results were also reflected at protein expression as MYB-silencing reduced expression of both *EGFR* and *RELA* in MiaPaCa-shMYB cells, while an opposite effect was seen in MYB overexpressing BXPC3 cells (Fig. 5B left panel). The densitometric analysis of the bands revealed a 9.65 and 1.65 fold decrease in protein expression of EGFR and RELA, respectively, upon MYB-silencing in MiaPaCa cell while its overexpression resulted in 6.90 and 1.49 fold increase in expression of these proteins (Fig. 5B, right panel). Moreover, alteration of MYB-expression in the PC cells significantly altered NF-κB transcriptional activity and was observed to directly correlate with the expression of MYB (Fig. 5C).

To examine if EGFR and RELA are direct transcriptional targets of MYB, we performed *in silico* analysis of ~1 kilobase DNA sequence 5′-upstream of the coding sequence (RefSeq ID NM_005228 and NM_021975, respectively) using online tools (ALGGEN-PROMO, TFBind). Two putative MYB binding sites were predicted in *RELA* promoter region (at −386 and −510 bases with respect to the first codon) and three potential binding sites for MYB were predicted in the promoter region of *EGFR* (at −499, −644 and −713 with respect to the first codon) (Fig. 5D). The direct binding of MYB to *EGFR* and *RELA* promoters was confirmed by ChIP assay. Pull-down of the *EGFR* and *RELA* promoter sequence was significantly decreased in MYB-silenced MiaPaCa-shMYB cells while opposite results were seen in MYB-overexpressing BxPC3 cells (Fig. 5E). Taken together, our data demonstrate that MYB enhances the expression of EGFR and RELA in PC by directly binding to their promoter region.

## Discussion

We recently identified MYB as an important regulator of pancreatic cancer pathogenesis which modulates tumor growth, aggressiveness as well as metastasis^6^. The analysis of the RNA-seq data of MYB-silenced MiaPaCa PC cells identified known MYB-target genes, e.g., *ADM, BRCA1, CBFB, KLF4, LDHA, PCNA* etc.^6^,^17^,^18^,^19^, along with several novel direct/indirect targets. These modulated genes belong to various protein families serving as cytokines, cellular transporters, enzymes, cell cycle regulators, DNA damage and repair molecules, transcription regulators etc. (Supplementary Tables 1 and 2). Being a transcription factor, silencing of MYB was expected to down-regulate a number of genes^17^,^18^,^19^, however, a simultaneous up-regulation of genes was also observed indicating a potential direct/indirect transcription repressor activity of MYB.

IPA analysis is a powerful tool to study interactions between molecular factors. IPA-network analysis illustrates the biological relationships between genes in a dataset at the molecular level. In this study, the Network 1 (Fig. 2A) suggested down-regulation of several proteins involved in alternative splicing of genes. The SRSF proteins depicted in this network have been demonstrated to have oncogenic potential and are frequently over expressed in many types of human tumors^20^,^21^. Similarly, *MALAT1* (metastasis associated lung adenocarcinoma transcript 1); a long non-coding RNA frequently overexpressed in PC^22^ was also decreased upon MYB-silencing. *MALAT1* has been demonstrated to act as transcriptional regulator of genes involved in cell growth, migration and invasion^23^. Apart from splicing factors, down-regulation of *DDX39A* (DEAD box RNA helicase 39A), a RNA helicase, that plays a role in RNA splicing/export was also observed. *DDX39A* up-regulation in lung squamous cell cancer has been demonstrated to promote tumor growth^24^. Indeed this network holds significant importance as the spliceosome is now being widely acknowledged as a novel target of oncogenic stress in cancers^25^,^26^. Alternative splicing and the differential expression of splicing factors has been reported in pancreatic cancer as well as other cancers to produce aberrant variants that facilitate tumor associated characteristics^27^,^28^,^29^. However, the factors governing aberrant splicing in cancers are largely unknown. There is evidence to suggest role of MYB in regulation of alternative splicing in normal hematopoietic cells^30^, but its role in cancer remains to be explored. Overall, the down-regulation of several oncogenic factors that are represented in the network 1, upon MYB silencing, supports the oncogenic potential of MYB.

The second network identified genes around the EGFR, GINS complex, MCM complex and primase nodes. EGFR, also known as ErbB1, is encoded by the *c*-*ERBB-1* a proto-oncogene and is a transmembrane tyrosine kinase growth factor receptor overexpressed in pancreatic cancer^31^,^32^. Down-regulation of GINS complex subunits (GINS1-4) is observed in our dataset (Fig. 2(B) and Supplementary Table 1). The PSF1/GINS1 is reported to be over-expressed in breast tumor cells as well as in highly proliferating cells where it enhances cell proliferation *via* increased DNA replication and anchorage independent growth of breast cancer cells^33^,^34^. The other node in this network includes mini chromosome maintenance complex (*MCM4, MCM5, MCM10,* and *MCMBP*). The MCM complex is over-expressed in human cancers and also in pre-cancerous cells undergoing malignant transformations. Its deregulation results in chromosomal defects and contributes to tumorigenesis^35^. Another axis involves the enzymes or proteins involved in DNA replication such as *POLA1*, encoding the catalytic subunit of DNA polymerase, which plays an essential role in DNA replication. *Prim1* and *Prim2* encoding smaller and larger subunit of DNA primase respectively form heterodimer that functions as a DNA-directed RNA polymerase to synthesize small RNA primers that are used to create okazaki fragments on the lagging strand of the DNA.

The third-top network identified upon silencing of MYB centered majorly around the down-regulation of NF-κB complex node. Constitutive activation of NF-κB transcriptional factors is observed in pancreatic cancers and regulates tumor development and progression. RIPK4 expression is also down-regulated in this group. It activates the NF-κB signaling^36^ and is also an important regulator of Wnt/beta-catenin signaling^37^, thus facilitating tumor growth and proliferation. Silencing of RIPK4 in cervical cancer cells inhibits cell migration and invasion through down-regulation of vimentin, MMP2 and fibronectin^37^. Interestingly, *MIB2* (mindbomb homolog 2, also known as skeletrophin) is one of the genes upregulated in this network. This gene encodes a RING (Really Interesting New Gene) finger-dependent ubiquitin ligase, and acts as a negative regulator of invasion in many malignant tumors through down-regulation of the *Met* oncogene^38^. The second central node in this network was identified around *KIAA1524/CIP2A* gene and the encoded protein stabilizes the *Met* oncogene, promoting epithelial mesenchymal transition (EMT) through the increased expression of vimentin and snail proteins^39^. Moreover, CIP2A also associates with H-Ras activating MEK/ERK pathways to promote EMT^39^. The third central node in this network involves genes that are known to regulate DNA replication and recombination, i.e. TIPIN, Rfc complex and ATM/ATR complex.

In addition to the gene networks, genes modulated upon MYB-silencing were assigned to several canonical signaling pathways indicating that MYB regulates different pathways to exert its oncogenic potential in PC. Some of these enriched canonical signaling pathways corresponded to the DNA replication and repair pathways. For example, MYB can directly/indirectly modulate the expression of CDC6, CDC7 and MCM group of proteins that are required for the initiation of replication. Further, activation of cell cycle regulatory pathways responsible for either G1/S arrest or G2/M arrest was also observed. Interestingly, up-regulation of the p53 signaling pathway was identified upon silencing of MYB. Mutations in the p53 gene, considered to inactivate the transcription factor, have been observed in nearly all pancreatic cancers and are also present in the MiaPaCa PC cells. However, in the MYB-silenced dataset, an up-regulation of PUMA suggests an additional degree of p53-independent regulation of the tumor.

In MYB-silenced cells, the pancreatic adenocarcinoma signaling was also negatively regulated due to the inhibition of EGFR and NF-κB. Furthermore, we identified both *RELA* and *EGFR* to be the direct transcriptional targets of MYB. It is possible that their reported overexpression in PC is through the MYB-mediated increase in mRNA levels. Overexpression of *RELA* has been reported in pancreatic adenocarcinomas and correlated with the activation of NF-κB pathway^40^. Interestingly, while NF-κB-P65 can be activated in response to various cytokines, growth factors, activating mutations; EGFR-dependent activation of NF-κB signaling has also been reported in pancreatic cancer^41^. Moreover, NF-κB activation in response to EGFR-targeted therapy has been identified as an adaptive resistance mechanism, thereby limiting EGFR therapy^42^.

In summary, we report for the first time various molecular mechanisms regulated by MYB in pancreatic cancer on a systemic level. We performed RNA-Seq analysis in MiaPaCa cell line model to generate a general model of MYB-dependent pathways in pancreatic cancer. We then used IPA analysis to predict the gene networks and canonical pathways affected by MYB signaling. The effects on many critical genes were not only validated in MiaPaCa model, but also verified in a reciprocal model by forced-expression of MYB in non-expressing cell line, further strengthening the role of MYB in pancreatic cancer pathogenesis. Among the many promising factors from several key signaling pathways, we identified a novel mechanistic involvement of EGFR and NF-κB in the MYB signaling which needs to be further explored, particularly in the context of therapeutic targeting of MYB signaling in PC patients.

## Materials and Methods

### Cell lines, reagents and antibodies

Stable MYB knockdown (MiaPaCa-shMYB), MYB overexpressing (BxPC3-MYB) and their respective control cell lines; non-targeting scrambled (MiaPaCa-NTScr) and empty vector control (BxPC3-Neo), respectively; generated previously^6^ were used in the study and were maintained as described earlier^6^. All the cells were tested and determined to be free of mycoplasma routinely and prior to the beginning of any analysis. The following reagents were used: Roswell Park Memorial Institute medium-1640 (RPMI-1640; Thermo Scientific, Logan, UT) supplemented with 5% fetal bovine serum (FBS; Atlanta Biologicals, Lawrenceville, GA); penicillin (100 IU ml^−1^) and streptomycin (0.1 mg ml^−1^; Invitrogen, Carlsbad, CA) at 37  °C and 5% CO_2_. For immunoblot assay, primary antibodies were used at a dilution of 1:1000, unless noted, and all corresponding secondary antibodies were used at 1:2000 dilution, the following antibodies were used in this study: MYB (rabbit monoclonal; Epitomics, Burlingame, CA); Adrenomedullin (ADM, rabbit polyclonal), KLF4 (rabbit polyclonal), SHH (rabbit polyclonal) (Abcam, Cambridge, MA); ALDH1A1 (rabbit monoclonal), PUMA (rabbit polyclonal), LDHA (rabbit monoclonal), GLUT1 (rabbit monoclonal), HDAC5 (rabbit monoclonal) (Cell Signalling Technology, Beverly, MA) EGFR (mouse monoclonal), MDM2 (mouse monoclonal), normal rabbit IgG (Santa Cruz Biotechnology, Dallas, TX); mouse biotinylated anti-β-actin (1:20,000; Sigma-Aldrich, St. Louis MO) and horseradish peroxidase (HRP) labeled secondary antibodies (Santa Cruz Biotechnology). Protein signals were detected using SuperSignal West Femto Maximum sensitivity substrate kit (Thermo Scientific). Chromatin Immunoprecipitation assay was performed using a ChIP-IT enzymatic kit (ChIP) kit (Active Motif, Carlsbad, CA).

### Total RNA isolation

Total RNA was extracted using TRIzol reagent (Invitrogen) from pancreatic cancer cell lines. The integrity and quality of the RNA was checked by denaturing RNA gels and quantitated on the Nanodrop 1000 (Thermo Scientific).

### cDNA synthesis and quantitative reverse transcription-PCR (qRT-PCR)

Two μg of total RNA isolated from pancreatic cancer cells was used for cDNA synthesis using the High Capacity Complementary DNA Reverse Transcription Kit (Thermo Scientific) following manufacturer’s instructions. cDNA constructed was used as a template with SYBR Green Master Mix on an iCycler system (Bio-Rad, Hercules, CA) along with specific primer pair sets to perform qRT-PCR in 96-well plates. Supplementary Table 3 lists the sequences of the primers used, their respective Tm, and GC content. The thermal conditions employed for real-time PCR assays were as follows: cycle 1: 95 °C for 10 min, cycle 2 (×40): 95 °C for 10 sec and annealing temperature respective to each primer pair for 45 sec.

### RNA-sequencing and bioinformatics analysis

RNA-sequencing and bioinformatics analysis were performed at the Genomics Core Facility at the Heflin Center for Genetics, University of Alabama at Birmingham, as previously described^43^. In brief, polyA^+^ mRNA isolated from MiaPaCa-NT-Scr and MiaPaCa-shMYB cells was converted to cDNA using random primers. Thereafter, cDNA sequencing libraries were generated using TruSeq library generation kit (Illumina, San Diego, CA) and quantified by qPCR using Roche LightCycler 480 with the Kapa Biosystems kit for library quantification (Kapa Biosystems, Wilmington, MA). Sequencing was performed on the Illumina HiSeq 2500 platform employing latest versions of sequencing reagents and flow cells generating up to 300 gigabytes of sequence information per flow cell. TopHat was used to align the raw RNA-Seq fastq reads to the human hg19 genome using the short read aligner Bowtie^44^. Cufflinks (version1.3.0) was used to align reads from TopHat and assemble transcripts, estimate their abundances and test for differential expression and regulation. The resulting RNA-Seq data was submitted to NCBI’s Gene Expression Omnibus (GEO) database and is accessible through GEO Series accession number GSE61290 (http://www.ncbi.nlm.nih.gov/geo/query/acc.cgi?acc=GSE61290).

### Immunoblotting analysis

Immunoblotting was performed by the standard procedures as described earlier^6^. Cell lysates resolved on 10% polyacrylamide gels were transferred to PVDF membranes and subjected to immunodetection procedure using specific antibodies of interest and visualized using the SuperSignal West Femto Maximum sensitivity substrate kit (Thermo Scientific) on Bio-Rad image analyzer (Bio-Rad). ImageJ (imagej.nih.gov) software was used for densitometric analysis. The values were normalized to the expression of β-actin and the experiments were performed in triplicates.

### Ingenuity pathway analysis

Networks and canonical pathways were generated through the Ingenuity Pathway Analysis (IPA; Ingenuity Systems, Qiagen, http://www.ingenuity.com/products/ipa) on the list of differentially expressed genes with the cutoff p-value ≤0.05 and fold change ±1.5. Gene symbols were used as the identifier and the IPA based Ingenuity Knowledge Base was used as a reference to perform core analysis. Network algorithm was used to generate the network of genes based on their connectivity and were assigned a score based on the number of focus genes. Canonical pathway analyses identified pathways from the IPA library that were of significance to the dataset.

### NF-κB transcriptional activity assay

In order to study the transcriptional activity of NF-κB, pancreatic cancer cell lines grown in 6-well plates were transfected with 1 μg of NF-κB-*firefly* luciferase based promoter reporter plasmid (pGL4.32 [luc2P/NF-κB-RE/Hygro]) and 0.5 μg of control reporter plasmid containing *Renilla reniformis* luciferase gene downstream of the TK promoter (pRL-TK). After 48 h, the transfected cells were harvested in passive lysis buffer and luciferase activity was measured using the Dual Luciferase Assay System (Promega, Madison, WI).

### Chromatin immunoprecipitation (ChIP) assay

ChIP assay was performed using a ChIP-IT enzymatic kit as previously described^42^. Briefly, DNA-protein cross-linking was done with paraformaldehyde (37%) followed by enzymatic DNA shearing. Sheared DNA was then subjected to immunoprecipitation using anti-MYB or normal rabbit IgG (as control). Subsequently, cross-linking reversed, proteins digested with proteinase K and DNA isolated. PCR was performed using specific primers sets flanking MYB-binding promoter regions (Supplementary Table 3) and amplification products resolved on a 1.5% agarose gel and visualized using ethidium bromide staining. Input DNA (without immunoprecipitation) and normal IgG-precipitated DNA were used as positive and negative controls, respectively.

## Additional Information

**How to cite this article**: Azim, S. *et al.* Deep sequencing and *in silico* analyses identify MYB-regulated gene networks and signaling pathways in pancreatic cancer. *Sci. Rep.* **6**, 28446; doi: 10.1038/srep28446 (2016).

## Supplementary Material

Supplementary Information

## References

[b1] Siegel R. L., Miller K. D. & Jemal A. Cancer statistics, 2016. CA Cancer J.Clin. 66, 7–30 (2016). 26742998 10.3322/caac.21332

[b2] Garrido-Laguna I. & Hidalgo M. Pancreatic cancer: from state-of-the-art treatments to promising novel therapies. Nat.Rev.Clin.Oncol. 12, 319–334 (2015). 25824606 10.1038/nrclinonc.2015.53

[b3] Maitra A. & Hruban R. H. Pancreatic cancer. Annu.Rev.Pathol. 3, 157–88, 157–188 (2008). 18039136 10.1146/annurev.pathmechdis.3.121806.154305 PMC2666336

[b4] Wallrapp C. *et al.* Characterization of a high copy number amplification at 6q24 in pancreatic cancer identifies c-myb as a candidate oncogene. Cancer Res. 57, 3135–3139 (1997). 9242439

[b5] Ramsay R. G. & Gonda T. J. MYB function in normal and cancer cells. Nat.Rev.Cancer. 8, 523–534 (2008). 18574464 10.1038/nrc2439

[b6] Srivastava S. K. *et al.* MYB is a novel regulator of pancreatic tumour growth and metastasis. Br.J.Cancer. 113, 1694–1703 (2015). 26657649 10.1038/bjc.2015.400 PMC4701995

[b7] Srivastava S. K. *et al.* Myb overexpression overrides androgen depletion-induced cell cycle arrest and apoptosis in prostate cancer cells and confers aggressive malignant traits: potential role in castration resistance. Carcinogenesis. 33, 1149–1157 (2012). 22431717 10.1093/carcin/bgs134 PMC3514863

[b8] Cheasley D. *et al.* Defective Myb Function Ablates Cyclin E1 Expression and Perturbs Intestinal Carcinogenesis. Mol.Cancer Res. 13, 1185–1196 (2015). 25934694 10.1158/1541-7786.MCR-15-0014

[b9] Drabsch Y., Robert R. G. & Gonda T. J. MYB suppresses differentiation and apoptosis of human breast cancer cells. Breast Cancer Res. 12, R55 (2010). 20659323 10.1186/bcr2614 PMC2949644

[b10] Persson M. *et al.* Recurrent fusion of MYB and NFIB transcription factor genes in carcinomas of the breast and head and neck. Proc.Natl.Acad.Sci.USA 106, 18740–18744 (2009). 19841262 10.1073/pnas.0909114106 PMC2773970

[b11] Stenman G., Persson F. & Andersson M. K. Diagnostic and therapeutic implications of new molecular biomarkers in salivary gland cancers. Oral Oncol. 50, 683–690 (2014). 24856188 10.1016/j.oraloncology.2014.04.008

[b12] Xia J. *et al.* NGS catalog: A database of next generation sequencing studies in humans. Hum.Mutat. 33, E2341–E2355 (2012). 22517761 10.1002/humu.22096 PMC4431973

[b13] Wang Z., Gerstein M. & Snyder M. RNA-Seq: a revolutionary tool for transcriptomics. Nat.Rev.Genet. 10, 57–63 (2009). 19015660 10.1038/nrg2484 PMC2949280

[b14] Patnala R., Clements J. & Batra J. Candidate gene association studies: a comprehensive guide to useful in silico tools. BMC.Genet. 14, 39, doi: 10.1186/1471-2156-14-39.,39-14 (2013). 23656885 PMC3655892

[b15] Calvano S. E. *et al.* A network-based analysis of systemic inflammation in humans. Nature. 437, 1032–1037 (2005). 16136080 10.1038/nature03985

[b16] Ficenec D. *et al.* Computational knowledge integration in biopharmaceutical research. Brief.Bioinform. 4, 260–278 (2003). 14582520 10.1093/bib/4.3.260

[b17] Lang G. *et al.* Myb proteins regulate the expression of diverse target genes. Oncogene. 24, 1375–1384 (2005). 15608679 10.1038/sj.onc.1208301

[b18] Lorenzo P. I. *et al.* Identification of c-Myb Target Genes in K562 Cells Reveals a Role for c-Myb as a Master Regulator. Genes Cancer 2, 805–817 (2011). 22393465 10.1177/1947601911428224 PMC3278898

[b19] Quintana A. M. *et al.* Dramatic repositioning of c-Myb to different promoters during the cell cycle observed by combining cell sorting with chromatin immunoprecipitation. PLoS.One. 6, e17362 (2011). 21364958 10.1371/journal.pone.0017362 PMC3043100

[b20] Fu Y. *et al.* SRSF1 and SRSF9 RNA binding proteins promote Wnt signalling-mediated tumorigenesis by enhancing beta-catenin biosynthesis. EMBO Mol.Med. 5, 737–750 (2013). 23592547 10.1002/emmm.201202218 PMC3662316

[b21] Gautrey H. L. & Tyson-Capper A. J. Regulation of Mcl-1 by SRSF1 and SRSF5 in cancer cells. PLoS.One. 7, e51497 (2012). 23284704 10.1371/journal.pone.0051497 PMC3524227

[b22] Pang E. J., Yang R., Fu X. B. & Liu Y. F. Overexpression of long non-coding RNA MALAT1 is correlated with clinical progression and unfavorable prognosis in pancreatic cancer. Tumour.Biol. 36, 2403–2407 (2015). 25481511 10.1007/s13277-014-2850-8

[b23] Jiao F. *et al.* Elevated expression level of long noncoding RNA MALAT-1 facilitates cell growth, migration and invasion in pancreatic cancer. Oncol.Rep. 32, 2485–2492 (2014). 25269958 10.3892/or.2014.3518

[b24] Sugiura T., Nagano Y. & Noguchi Y. DDX39, upregulated in lung squamous cell cancer, displays RNA helicase activities and promotes cancer cell growth. Cancer Biol.Ther. 6, 957–964 (2007). 17548965 10.4161/cbt.6.6.4192

[b25] Koh C. M., Sabo A. & Guccione E. Targeting MYC in cancer therapy: RNA processing offers new opportunities. Bioessays. 10 (2016). 10.1002/bies.201500134 PMC4819695 26778668

[b26] Hsu T. Y. *et al.* The spliceosome is a therapeutic vulnerability in MYC-driven cancer. Nature. 525, 384–388 (9-17-2015). 26331541 10.1038/nature14985 PMC4831063

[b27] Shkreta L. *et al.* Cancer-Associated Perturbations in Alternative Pre-messenger RNA Splicing. Cancer Treat.Res. 158, 41–94 (2013). 24222354 10.1007/978-3-642-31659-3_3

[b28] Siddiqui A. A. *et al.* Osteopontin splice variant as a potential marker for metastatic disease in pancreatic adenocarcinoma. J. Gastroenterol. Hepatol. 29, 1321–1327 (2014). 24548099 10.1111/jgh.12561 PMC4465289

[b29] Arafat H. *et al.* Tumor-specific expression and alternative splicing of the COL6A3 gene in pancreatic cancer. Surgery. 150, 306–315 (2011). 21719059 10.1016/j.surg.2011.05.011 PMC3163121

[b30] Orvain C., Matre V. & Gabrielsen O. S. The transcription factor c-Myb affects pre-mRNA splicing. Biochem.Biophys.Res.Commun. 372, 309–313 (2008). 18498763 10.1016/j.bbrc.2008.05.054

[b31] Friess H. *et al.* Growth factor receptors are differentially expressed in cancers of the papilla of vater and pancreas. Ann.Surg. 230, 767–774 (1999). 10615931 10.1097/00000658-199912000-00005 PMC1420940

[b32] Tzeng C. W. *et al.* EGFR genomic gain and aberrant pathway signaling in pancreatic cancer patients. J.Surg.Res. 143, 20–26 (2007). 17950068 10.1016/j.jss.2007.01.051

[b33] Nakahara I. *et al.* Up-regulation of PSF1 promotes the growth of breast cancer cells. Genes Cells. 15, 1015–1024 (2010). 20825491 10.1111/j.1365-2443.2010.01442.x

[b34] MacNeill S. A. Structure and function of the GINS complex, a key component of the eukaryotic replisome. Biochem.J. 425, 489–500 (2010). 20070258 10.1042/BJ20091531

[b35] Lei M. The MCM complex: its role in DNA replication and implications for cancer therapy. Curr.Cancer Drug Targets. 5, 365–380 (2005). 16101384 10.2174/1568009054629654

[b36] Holland P. *et al.* RIP4 is an ankyrin repeat-containing kinase essential for keratinocyte differentiation. Curr.Biol. 12, 1424–1428 (2002). 12194825 10.1016/s0960-9822(02)01075-8

[b37] Liu D. Q. *et al.* Increased RIPK4 expression is associated with progression and poor prognosis in cervical squamous cell carcinoma patients. Sci.Rep. 5, 11955, doi: 10.1038/srep11955., 11955 (2015). 26148476 PMC4493702

[b38] Takeuchi T. *et al.* A ubiquitin ligase, skeletrophin, is a negative regulator of melanoma invasion. Oncogene. 25, 7059–7069 (2006). 16715130 10.1038/sj.onc.1209688

[b39] Wu Y., Gu T. T. & Zheng P. S. CIP2A cooperates with H-Ras to promote epithelial-mesenchymal transition in cervical-cancer progression. Cancer Lett. 356, 646–655 (2015). 25458953 10.1016/j.canlet.2014.10.013

[b40] Weichert W. *et al.* High expression of RelA/p65 is associated with activation of nuclear factor-kappaB-dependent signaling in pancreatic cancer and marks a patient population with poor prognosis. Br.J.Cancer. 97, 523–530 (2007). 17622249 10.1038/sj.bjc.6603878 PMC2360349

[b41] De S., Dermawan J. K. & Stark G. R. EGF receptor uses SOS1 to drive constitutive activation of NFkappaB in cancer cells. Proc.Natl.Acad.Sci.USA 111, 11721–11726 (2014). 25071181 10.1073/pnas.1412390111 PMC4136585

[b42] Arora S. *et al.* An undesired effect of chemotherapy: gemcitabine promotes pancreatic cancer cell invasiveness through reactive oxygen species-dependent, nuclear factor kappaB- and hypoxia-inducible factor 1alpha-mediated up-regulation of CXCR4. J.Biol.Chem. 288, 21197–21207 (2013). 23740244 10.1074/jbc.M113.484576 PMC3774385

[b43] Jiang W. *et al.* WNT5A inhibits metastasis and alters splicing of Cd44 in breast cancer cells. PLoS.One. 8, e58329 (2013). 23484019 10.1371/journal.pone.0058329 PMC3590134

[b44] Langmead B., Trapnell C., Pop M. & Salzberg S. L. Ultrafast and memory-efficient alignment of short DNA sequences to the human genome. Genome Biol. 10, R25–10 (2009). 19261174 10.1186/gb-2009-10-3-r25 PMC2690996

