# Supplementary material for: Deep sequencing and in silico analyses identify MYB-regulated gene networks and signaling pathways in pancreatic cancer
Source: Sci Rep. 2016 Jun 29;6:28446. doi: 10.1038/srep28446 (PMC4926062; doi:10.1038/srep28446)
Supplement: Supplementary Information [file srep28446-s1.pdf]

Deep sequencing and *in silico* analyses identify MYB-regulated gene networks and signaling pathways in pancreatic cancer

Shafquat Azim[1†], Haseeb Zubair[1†], Sanjeev K Srivastava[1], Arun Bhardwaj[1], Asif Zubair[2], Aamir Ahmad[1], Seema Singh[1,3], Moh'd Khushman[4], Ajay P Singh[1,3*]

[1]*Department of Oncologic Sciences, Mitchell Cancer Institute, University of South Alabama, Mobile, AL 36604, USA*
[2]*Molecular and Computational Biology, School of Biological Sciences, Dornslife College of Arts Letters and Sciences, University of Southern California, Los Angeles, CA 90089, USA*
[3]*Department of Biochemistry and Molecular Biology, College of Medicine, University of South Alabama, Mobile, AL 36688, USA*
[4]*Department of Interdisciplinary Clinical Oncology, Mitchell Cancer Institute, University of South Alabama, Mobile, AL 36604, USA*

Supplementary Information

[*]To whom correspondence should be addressed:

Ajay P. Singh, Ph.D.
Department of Oncologic Sciences
Mitchell Cancer Institute
University of South Alabama
1660 Springhill Avenue
Mobile, AL 36604-1405, U.S.A.
Phone: 251-445-9843; Fax: 251-460-6994
E-mail: asingh@health.southalabama.edu

Supplementary Table 1: List of genes modulated upon MYB-silencing in MiaPaCa cell line with fold change ≥ ± 1.5 and p-value ≤ 0.05

**Exp Fold Change**	**Exp p-value**	**Entrez Gene Name**
-1.719	3.74E-02	AASDHPPT
-1.708	3.72E-02	AATF
1.93	1.70E-02	ABCA7
4.266	4.40E-03	ABCC3
-1.852	2.96E-02	ABCC4
5.283	1.00E-04	ABCG1
2.278	4.23E-02	ABHD16B
1.95	4.54E-02	ABHD8
-1.752	3.84E-02	ABLIM1
2.078	1.55E-02	ABTB1
-2.323	9.95E-03	ABTB2
2.209	8.50E-03	ACADS
1.949	2.15E-02	ACADVL
-2.95	1.13E-02	ACOX2
4.323	1.65E-03	ADAMTS4
2.412	1.67E-02	ADCY5
-1.647	5.00E-02	ADI1
-5.061	2.70E-02	ADM
-2.734	1.71E-02	ADORA2A
-1.866	2.66E-02	AEN
-1.709	3.96E-02	AGGF1
1.951	1.27E-02	AGRN
-2.092	4.55E-03	AK4
2.08	1.35E-02	AKAP12
-3.285	5.00E-05	ALDH1A1
1.752	2.78E-02	ALDH1A3
2.071	7.10E-03	ALDH3B1
-2.746	5.00E-05	ALDH9A1
-2.798	5.75E-03	ALDOC
-2.192	1.87E-02	ALG10
-2.034	1.38E-02	ALG10B
-5.145	9.00E-04	ALOXE3
-1.692	4.30E-02	ALYREF
-1.768	4.23E-02	AMMECR1
-1.804	2.60E-02	AMOTL2
-6.314	3.35E-02	ANKRD1
-1.987	2.34E-02	ANKRD26
-1.697	3.94E-02	ANKRD28

-1.843	4.13E-02	ANKRD32
-3.424	4.28E-02	ANKRD5
2.606	1.18E-02	ANKRD9
-2.08	7.30E-03	ANLN
-2.046	9.60E-03	ANXA1
-2.857	2.43E-02	ANXA10
-1.662	4.53E-02	AP3M1
1.814	4.40E-02	APLP1
-1.749	2.95E-02	ARHGAP12
-1.745	4.33E-02	ARHGAP18
5.177	2.15E-03	ARHGAP31
-1.848	1.96E-02	ARHGAP32
-1.763	3.67E-02	ARID1A
1.748	4.86E-02	ARRDC2
-2.03	6.95E-03	ARRDC3
1.847	3.84E-02	ARSA
1.874	1.75E-02	ASNS
-1.891	1.60E-02	ATAD2
-2.226	1.09E-02	ATAD5
-2.592	2.55E-03	ATF3
1.855	3.86E-02	ATHL1
1.709	3.96E-02	ATP13A2
1.964	1.00E-02	ATP8B2
-2.869	9.60E-03	AUNIP
-1.722	3.87E-02	BAG3
2.622	4.00E-03	BAI2
-1.971	2.31E-02	BARD1
-1.915	2.38E-02	BARX2
2.26	1.09E-02	BBC3
-1.706	3.82E-02	BCAR1
2.143	2.41E-02	BCAT1
2.206	1.06E-02	BCL7A
-2.326	1.25E-03	BCLAF1
-4.773	3.01E-02	BCO2
2.197	4.22E-02	BEAN1
-5.805	5.00E-05	BIRC3
3.238	8.90E-03	BIRC7
-2.388	8.50E-03	BLM
1.856	1.96E-02	BLVRB
2.513	2.94E-02	BMF
-1.777	2.43E-02	BMS1

-1.922	1.26E-02	BNIP3L
-1.783	3.17E-02	BRCA1
-2.206	7.25E-03	BRCA2
-1.866	1.87E-02	BRCC3
-1.742	4.38E-02	BRE
-1.85	2.47E-02	BTF3
-2.094	1.40E-02	BTG2
-2.693	2.03E-02	BTN2A3P
-2.079	2.92E-02	C11orf82
1.81	2.92E-02	C12orf57
-3.652	2.88E-02	C15orf48
2.037	2.25E-02	C17orf103
2.002	4.22E-02	C19orf26
-3.774	5.00E-05	C1orf110
-2.239	1.24E-02	C1orf226
2.196	1.76E-02	C5orf4
2.447	3.15E-03	C6orf1
-2.093	3.65E-02	C8orf46
2.853	1.62E-02	CACNA2D2
2.121	1.27E-02	CACNB3
-2.21	4.40E-03	CASP2
-4.057	5.00E-05	CAV1
-1.806	1.98E-02	CBFB
-1.678	4.43E-02	CCAR1
-1.747	4.40E-02	CCDC137
-2.515	1.70E-03	CCDC58
-2.105	4.40E-03	CCDC68
-1.923	1.53E-02	CCNF
-2.485	6.25E-03	CCRN4L
-1.779	2.91E-02	CD109
1.707	4.08E-02	CD9
-2.374	2.05E-02	CDA
-1.92	3.17E-02	CDC25A
-2.15	5.30E-03	CDC6
-1.837	3.24E-02	CDC7
-2.022	9.30E-03	CDCA5
-1.721	3.30E-02	CDCA7
-1.956	1.11E-02	CDK1
-1.855	2.57E-02	CDK2
-3.886	5.00E-05	CDK6
-3.599	1.75E-03	CDKN1A

1.801	3.30E-02	CEBPB
-1.707	3.21E-02	CENPE
-1.718	3.43E-02	CEP55
1.841	3.87E-02	CFD
-1.715	4.86E-02	CHAF1B
-1.867	2.53E-02	CHAMP1
1.862	1.53E-02	CHD3
-1.746	3.66E-02	CHEK1
-2.71	4.00E-04	CHORDC1
2.133	3.60E-03	CHPF
-1.748	3.91E-02	CHRAC1
2.806	7.90E-03	CHRD
2.347	3.30E-02	CHRM4
2.891	2.33E-02	CHST11
-1.834	2.97E-02	CHST3
-2.991	1.50E-04	CITED2
-1.698	3.84E-02	CKAP2
-1.788	3.48E-02	CKAP2L
-1.808	2.40E-02	CKS1B
1.664	4.88E-02	CLIP2
1.965	1.55E-02	CLIP4
-1.763	3.12E-02	CLNS1A
-1.956	1.41E-02	CLOCK
-3.289	5.00E-05	CLSPN
-1.771	3.37E-02	CMSS1
1.853	2.04E-02	COL18A1
1.725	3.99E-02	COL4A2
2.427	9.00E-04	COL6A2
-1.703	3.93E-02	COPS3
-2.054	4.21E-02	CPSF1
3.837	1.14E-02	CREB3L3
2.416	1.70E-03	CREBRF
2.152	2.22E-02	CRELD1
2.258	4.25E-03	CRELD2
1.731	3.55E-02	CRLF1
-1.664	4.64E-02	CTNNAL1
-1.829	3.43E-02	CTSC
1.867	1.86E-02	CTSL1
1.742	2.96E-02	CUL7
-1.844	3.12E-02	CWF19L1
-4.886	1.38E-02	CXCL2

-2.048	1.27E-02	CXCL5
1.699	4.21E-02	CYBA
2.097	3.72E-02	CYS1
-1.78	2.50E-02	DARS
1.786	3.62E-02	DBP
-2.257	6.90E-03	DCLRE1B
-2.124	6.55E-03	DDR2
-1.81	4.20E-02	DDX11
-1.805	2.67E-02	DDX39A
-1.697	4.61E-02	DDX41
-2.051	3.91E-02	DEM1
-1.76	2.81E-02	DEPDC1
-25.269	4.28E-02	DES
-1.911	1.73E-02	DHX9
-5.186	1.00E-03	DKK1
2.285	3.87E-02	DLL1
-2.078	1.58E-02	DNA2
-1.753	3.26E-02	DNAJA1
-1.85	2.94E-02	DNAJC9
1.773	3.06E-02	DNASE2
-1.851	2.22E-02	DNMBP
-1.874	4.21E-02	DPCD
-1.953	9.35E-03	DROSHA
-2.095	3.05E-02	DTL
1.822	2.08E-02	DVL1
-2.464	9.20E-03	E2F8
1.756	3.89E-02	ECI1
-1.824	4.83E-02	EED
1.73	3.86E-02	EGFL7
-2.214	2.55E-03	EGFR
1.822	2.26E-02	EGR1
2.61	1.29E-02	EGR3
-2.169	2.80E-03	EHF
-1.674	4.40E-02	EI24
-1.67	4.98E-02	EIF3M
-1.724	3.52E-02	EIF4EBP2
1.761	3.08E-02	ELFN1
-1.033	2.73E-02	ENO1-AS1
2.412	1.58E-02	EPAS1
-2.817	3.60E-02	EPB41L4B
-1.755	4.61E-02	EPC1

-2.32	8.45E-03	ERCC6L
1.941	3.18E-02	ERO1LB
-3.395	3.50E-04	ESCO2
-1.821	4.50E-02	ESRG
-1.869	2.63E-02	ETS1
-2.384	3.50E-03	EXO1
-1.826	2.28E-02	EXOSC2
-2.042	7.45E-03	EZH2
-2.028	1.70E-02	FAIM
-3.778	2.00E-04	FAM111B
2.803	5.50E-04	FAM129A
-1.958	9.80E-03	FAM136A
-2.373	3.55E-03	FAM13B
4.404	3.15E-02	FAM149A
-1.854	3.01E-02	FAM162A
3.807	4.76E-02	FAM171B
-2.531	9.00E-03	FAM173B
2.126	3.84E-02	FAM176B
-2.531	1.23E-02	FAM198B
-10.342	4.93E-02	FAM212A
-1.994	4.96E-02	FAM54A
-2.474	8.55E-03	FAM5C
-2.725	2.95E-03	FAM9C
-1.95	3.53E-02	FANCM
-1.707	4.00E-02	FARP2
1.764	3.52E-02	FBXL16
-2.068	1.00E-02	FBXO5
2.177	7.45E-03	FBXW4
2.029	2.58E-02	FCGRT
-1.886	2.62E-02	FEM1C
-2.158	7.65E-03	FEN1
-2.792	4.60E-02	FGF5
-1.986	1.55E-02	FGFR1OP
2.438	6.15E-03	FHL1
2.421	3.45E-02	FIBCD1
-2.145	1.08E-02	FIGNL1
2.535	1.56E-02	FNDC4
-2.498	3.50E-04	FOSL1
2.481	2.40E-02	FRS3
-1.942	8.30E-03	FRYL
1.793	2.78E-02	FURIN

-2.698	1.50E-04	FUS
2.717	2.15E-02	FYN
-1.844	2.31E-02	FZD4
-1.822	2.81E-02	G3BP1
2.419	5.55E-03	GABARAPL1
-2.659	6.50E-04	GADD45A
3.437	2.25E-02	GALNTL1
2.284	4.03E-02	GATA2
3.464	1.50E-04	GATSL3
2.456	1.74E-02	GBX2
3.729	3.50E-04	GDF15
2.419	4.70E-02	GDPD1
-2.306	3.50E-03	GEN1
-1.937	1.57E-02	GINS1
-1.996	1.92E-02	GINS2
-2.014	1.37E-02	GINS3
-1.775	2.43E-02	GINS4
-1.896	1.28E-02	GJC1
5.113	6.55E-03	GLDN
2.259	1.67E-02	GNAO1
-2.687	3.74E-02	GNG2
2.117	4.40E-03	GPC1
1.865	4.83E-02	GPR137B
1.716	3.86E-02	GRAMD1A
2.188	4.21E-02	GRAMD1B
3.479	5.00E-05	GRB7
-1.77	3.82E-02	GRPEL1
-1.726	3.94E-02	GSPT1
-2.155	4.15E-03	GTF2A1
-1.9	1.65E-02	GTF3C6
2.222	2.88E-02	HAGHL
2.058	7.35E-03	HCN2
1.899	2.27E-02	HDAC5
-2.689	3.00E-04	HDAC9
11.707	2.50E-04	HEATR6
1.842	4.37E-02	HECA
2.435	2.31E-02	HECW1
-2.527	2.25E-03	HELLS
1.873	1.64E-02	HELZ2
-1.82	4.91E-02	HENMT1
1.919	1.08E-02	HERPUD1

2.128	2.15E-02	HES4
5.375	2.09E-02	HES7
-4.461	5.00E-05	HIF1A
-4.13	3.33E-02	HIF1A-AS2
-1.853	1.54E-02	HIGD1A
-7.523	4.47E-02	HIST1H1B
-4.451	3.96E-02	HIST1H1E
-8.682	3.60E-02	HIST2H2BF
-1.735	3.28E-02	HK2
-2.111	1.28E-02	HKDC1
2.175	4.49E-02	HLA-A
5.045	4.30E-03	HLA-B
2.345	4.79E-02	HLA-C
-1.914	2.23E-02	HMGB1
-1.708	4.33E-02	HMGB3
-1.777	2.40E-02	HMMR
3.481	5.00E-05	HMOX1
-1.68	4.42E-02	HNRNPD
-1.756	3.06E-02	HNRNPH3
-2.092	4.80E-03	HNRNPM
-1.823	4.33E-02	HNRNPU
2.111	5.20E-03	HOXB8
2.774	1.50E-04	HOXB9
-1.87	2.77E-02	HSP90AB2P
2.241	1.02E-02	HSPA5
-1.75	3.83E-02	HSPH1
1.719	3.45E-02	HYOU1
2.371	1.05E-03	ICAM5
3.323	2.40E-02	ID4
2.582	4.16E-02	IDUA
1.694	4.85E-02	IER5L
2.239	1.52E-02	IFI27L2
-2.167	6.25E-03	IFIT1
-2.306	7.15E-03	IFIT3
-2.261	8.05E-03	IFLTD1
-1.837	4.45E-02	IFT172
2.025	8.10E-03	IGFBP6
1.931	4.47E-02	IGSF8
-3.163	6.40E-03	IKZF2
2.083	1.54E-02	ILF3-AS1
-1.935	1.41E-02	INPP4B

1.744	3.38E-02	INSIG1
-2.216	4.46E-02	IQCC
-1.681	4.76E-02	IQCE
1.754	3.41E-02	IRF2BPL
-3.275	2.85E-02	ISPD
1.732	4.44E-02	ITGA5
1.811	3.01E-02	ITGB4
1.736	4.66E-02	JMY
2.154	3.95E-03	JUND
-1.748	2.86E-02	KAT6B
2.255	4.21E-02	KCNAB2
7.627	6.55E-03	KCNJ4
6.492	2.25E-02	KCNK3
4.687	3.19E-02	KCP
-1.851	2.09E-02	KCTD9
1.779	3.87E-02	KDM6B
2.456	2.25E-02	KIAA1211
2.121	2.46E-02	KIAA1244
-1.706	4.63E-02	KIAA1279
-1.91	4.56E-02	KIAA1524
-1.9	1.29E-02	KIF11
-2.077	7.60E-03	KIF18A
1.962	9.15E-03	KIF1A
-2.008	8.35E-03	KIF20B
-1.836	1.79E-02	KIF23
-1.893	1.54E-02	KIF4A
1.963	2.67E-02	KIFC2
-2.062	4.15E-02	KLF4
2.478	1.65E-03	KLF9
2.053	3.91E-02	KLHL17
2.331	8.00E-04	KLHL24
-1.782	2.45E-02	KNTC1
2.32	1.14E-02	KREMEN2
-2.056	4.54E-02	KRT13
-11.957	5.65E-03	KRT16
-1.85	3.05E-02	KRT80
-4.133	5.00E-05	LDHA
1.745	3.69E-02	LENG8
1.906	1.54E-02	LGALS3BP
1.978	4.25E-02	LHX1
-1.679	4.86E-02	LIN7C

-2.234	9.50E-03	LIN9
-1.707	4.08E-02	LIPA
-1.907	3.35E-02	LLPH
1.688	4.91E-02	LMF2
-2.065	6.50E-03	LMNB1
1.993	3.30E-02	LMO7
3.662	4.10E-02	LOC344887
-2.238	5.00E-03	LOC389831
-2.184	1.76E-02	LOC441666
-4.432	2.89E-02	LOC646736
1.983	9.25E-03	LPCAT3
-2.462	4.05E-03	LPXN
2.409	2.10E-03	LRP10
1.919	1.46E-02	LRRC33
4.86	2.00E-04	LTBP1
2.377	3.70E-03	LTBP4
-1.828	1.96E-02	LUC7L3
-1.936	1.58E-02	LYAR
2.032	1.58E-02	MACROD1
-1.911	2.65E-02	MAGOHB
-1.749	2.99E-02	MALAT1
-1.708	4.02E-02	MALL
-2.005	4.78E-02	MAML2
2.163	3.55E-03	MAN2B1
1.731	4.97E-02	MANF
-2.678	2.46E-02	MAP2
-2.061	5.60E-03	MARCKSL1
-1.941	1.42E-02	MASTL
-4.147	1.81E-02	MB
-2.883	9.00E-04	MCM10
-1.896	1.73E-02	MCM2
-2.124	2.60E-03	MCM3
-2.23	2.05E-03	MCM4
-1.929	1.27E-02	MCM5
-1.836	1.90E-02	MCMBP
-2.306	5.85E-03	MCTP1
-1.751	3.02E-02	MCU
-2.296	1.60E-03	MDM2
2.027	1.66E-02	MEIS3
-1.722	3.65E-02	MELK
2.064	2.23E-02	MESDC1

3.263	1.60E-03	METRN
2.59	7.55E-03	MFSD3
1.718	4.68E-02	MIB2
2.27	6.30E-03	MICAL1
-2.914	2.86E-02	MIR137HG
-5.189	2.36E-02	MIR210HG
-1.764	3.90E-02	MKI67IP
2.35	2.40E-03	MKNK2
3.644	2.50E-03	MMP14
2.154	9.60E-03	MNT
-1.959	9.25E-03	MORF4L1
-1.856	2.36E-02	MPHOSPH9
2.145	3.45E-03	MRC2
-2.125	1.35E-02	MRPL1
2.012	4.30E-02	MSI1
-1.876	2.00E-02	MSL2
5.974	4.21E-02	MSLN
-2.017	3.66E-02	MT1A
-3.109	7.65E-03	MT1M
-2.626	2.09E-02	MTERF
1.73	4.49E-02	MVD
-1771.85	5.00E-05	MYB
-2.071	8.15E-03	MYBL1
-2.151	1.24E-02	MYH16
-15.566	3.50E-04	MYH7
-1.791	4.33E-02	MYOF
-2.751	7.00E-04	MYPN
4.046	1.15E-02	N4BP2L1
-1.867	2.91E-02	NAA15
2.087	1.67E-02	NAGLU
-1.655	4.93E-02	NAP1L4
-1.796	3.77E-02	NARF
1.814	4.71E-02	NAT14
-2.029	2.80E-02	NCAPG
-1.737	3.02E-02	NCAPG2
-1.852	3.57E-02	NCAPH
-1.746	4.88E-02	NCBP1
-1.9	2.82E-02	NCOA5
-1.914	1.41E-02	NDC80
2.039	8.50E-03	NEAT1
-1.675	4.14E-02	NET1

-4.251	3.19E-02	NEURL3
-2.068	4.75E-02	NFIB
2.842	4.47E-02	NIPAL4
-1.906	4.92E-02	NMI
2.688	1.12E-02	NOG
-1.862	1.62E-02	NOL11
-1.857	2.14E-02	NOL9
-2.25	1.45E-03	NOP58
-2.318	2.50E-02	NOS1AP
2.362	3.52E-02	NOXA1
-1.75	4.08E-02	NPAT
2.088	9.90E-03	NPDC1
-2.438	1.91E-02	NPIP
-2.275	1.65E-03	NR2F2
-3.14	7.00E-04	NRIP1
-3.631	6.45E-03	NRP1
-1.916	1.55E-02	NSRP1
-1.943	1.23E-02	NT5C2
-2.124	4.50E-03	NT5DC2
2.277	2.66E-02	NUDT18
-1.678	4.36E-02	NUP153
-1.711	3.58E-02	NUP155
-1.949	9.40E-03	NUP50
4.56	1.00E-04	NUPR1
-1.724	3.61E-02	NXF1
2.423	4.57E-02	NXPH4
-7.717	2.79E-02	OAS2
-2.688	1.27E-02	OASL
2.196	4.98E-02	OCEL1
-2.55	4.50E-04	ODC1
1.917	3.33E-02	OGDHL
3.35	7.00E-04	OLFM2
1.973	2.33E-02	ORAI3
-2.066	1.49E-02	ORC1
-1.812	3.50E-02	ORC6
-1.799	3.69E-02	OSBPL3
-2.535	9.60E-03	OSR1
-2.659	4.00E-02	P2RY2
-1.91	1.18E-02	P4HA1
-1.804	2.20E-02	PA2G4
-1.757	3.29E-02	PAICS

2.442	1.10E-03	PANX2
-1.768	3.45E-02	PARVA
2.118	4.85E-03	PBXIP1
-1.717	3.52E-02	PCF11
1.997	4.21E-02	PCK2
-1.835	1.90E-02	PCNA
-1.716	3.87E-02	PCNT
2.168	2.36E-02	PCSK1N
-1.703	4.30E-02	PDCD5
2.164	2.99E-02	PDGFB
-1.86	1.69E-02	PDGFC
1.708	4.40E-02	PDIA5
-2.562	1.35E-03	PDK1
8.891	4.00E-04	PDK4
-3.268	4.50E-04	PER2
-3.196	3.50E-04	PFKFB4
-2.265	2.50E-03	PGAP1
-1.872	2.65E-02	PGK1
1.857	1.74E-02	PGPEP1
-1.679	4.68E-02	PHAX
2.538	7.50E-04	PHGDH
-1.998	2.15E-02	PIGA
1.921	2.54E-02	PIGQ
-1.887	2.22E-02	PIK3R3
2.786	2.50E-04	PIM1
1.769	2.88E-02	PIM3
2.264	1.85E-03	PITPNM1
1.798	4.66E-02	PITX1
3.982	3.15E-03	PKD1L2
-3.502	3.90E-03	PLAC8
5.363	1.77E-02	PLAG1
-1.671	4.59E-02	PLAU
-4.358	2.80E-03	PLCXD3
1.946	1.30E-02	PLD3
-1.805	2.61E-02	PLEKHA2
1.727	3.33E-02	PLIN2
-1.734	3.34E-02	PLK1
1.691	4.94E-02	PLXNA3
-2.25	6.75E-03	PMEPA1
-1.79	2.50E-02	PNN
1.671	4.23E-02	PNPLA6

-1.861	1.48E-02	POLA1
-2.09	9.10E-03	POLD3
-2.609	9.30E-03	POLE2
-3.068	1.00E-04	POLQ
-1.884	3.43E-02	POLR3G
2.401	1.30E-02	PPIC
-1.871	2.05E-02	PPIF
-2.162	1.01E-02	PPM1D
-1.69	4.71E-02	PPME1
-2.287	2.65E-03	PPP1R15A
-2.177	4.20E-03	PPP1R2
1.656	4.67E-02	PPP1R26
-2.004	5.65E-03	PPP4R1
-1.711	3.72E-02	PPP6R3
1.732	4.55E-02	PRAF2
-2.264	2.32E-02	PRICKLE1
-1.904	4.78E-02	PRIM1
-2.184	9.35E-03	PRIM2
-6.571	2.12E-02	PRIMA1
-1.816	2.50E-02	PRMT3
1.745	4.83E-02	PRND
2.698	1.06E-02	PROCR
-1.699	4.59E-02	PRPF3
-1.737	3.19E-02	PRPF38B
-1.793	2.32E-02	PRPF4
-1.699	4.24E-02	PRPF4B
-1.815	1.97E-02	PRPS2
-2.696	1.00E-04	PRRG4
2.265	4.86E-02	PRSS16
-1.87	1.69E-02	PSMD12
-2.036	6.90E-03	PSPC1
2.007	4.24E-02	PTCH1
-2.061	7.00E-03	PTP4A1
2.001	1.40E-02	PTPRU
1.721	3.44E-02	PVRL2
-2.055	3.76E-02	PVT1
2.097	1.10E-02	QPCTL
-1.741	2.88E-02	QSER1
-1.693	4.71E-02	RAB22A
-1.776	3.19E-02	RAB31
1.853	4.89E-02	RAB32

2.604	6.50E-04	RABAC1
-2.457	1.11E-02	RAD51
-2.007	2.89E-02	RAD51C
-1.897	1.75E-02	RAPGEF6
-1.778	3.18E-02	RASAL2
2.093	8.05E-03	RBCK1
-2.287	3.10E-03	RBL1
-1.763	3.00E-02	RBM26
-1.839	2.26E-02	RBMX
-3.582	4.10E-03	RCBTB2
-4.638	5.00E-05	RELA
16.466	5.00E-05	REXO1
-2.553	3.00E-04	RFC3
-1.98	9.35E-03	RFWD3
-2.34	6.00E-03	RGS5
1.783	3.70E-02	RHPN1
-1.902	1.40E-02	RIF1
-3.395	2.60E-03	RIMKLA
-1.823	2.78E-02	RIN2
-1.807	3.17E-02	RIPK1
-1.991	2.62E-02	RIPK4
-1.705	4.54E-02	RNF138
-1.893	1.36E-02	RNF182
3.254	5.50E-04	RNF215
-2.926	3.86E-02	ROCK1P1
-1.861	4.53E-02	RPP14
1.969	1.04E-02	RPS21
2.453	4.83E-02	RPS6KA2
1.722	4.16E-02	RRAGD
2.241	5.70E-03	RRAS
2.198	1.29E-02	RTN4R
1.909	2.44E-02	S100A6
-1.797	2.20E-02	SAFB
-2.095	8.95E-03	SAFB2
-2.288	2.30E-03	SAT1
-1.948	1.98E-02	SBDSP1
2.884	2.04E-02	SCN1B
-2.424	7.45E-03	SDPR
3.423	2.00E-04	SEC24D
-2.217	2.10E-03	SEMA3A
-3.961	5.00E-05	SEMA3C

-4.5	7.50E-04	SEMA3D
2.505	1.80E-02	SERINC2
2.144	6.10E-03	SERPINA3
-2.385	2.30E-03	SESN2
1.974	8.25E-03	SEZ6L2
-1.721	4.74E-02	SF3B4
-3.491	2.21E-02	SFN
-2.316	1.95E-03	SFPQ
-2.103	4.90E-02	SFXN2
-2.117	5.05E-03	SGOL2
1.959	1.84E-02	SGSH
-2.52	4.25E-03	SH3RF2
2.081	8.70E-03	SHC1
2.522	2.98E-02	SHC2
-1.691	4.71E-02	SHCBP1
-2.493	3.15E-03	SHH
-1.669	4.83E-02	SKA2
-2.575	2.15E-02	SKA3
-1.903	1.26E-02	SKP1
2.01	1.40E-02	SLC16A2
1.99	2.39E-02	SLC16A5
5.584	7.05E-03	SLC17A7
1.96	2.83E-02	SLC20A1
2.695	9.95E-03	SLC22A17
2.257	8.65E-03	SLC22A18
-2.058	5.30E-03	SLC2A1
-2.445	8.50E-04	SLC30A1
-2.134	4.27E-02	SLC38A4
-2.63	3.00E-04	SLC39A10
1.875	1.67E-02	SLC45A1
1.879	3.19E-02	SLC6A6
2.917	1.25E-03	SLC9A3
2.581	2.26E-02	SLC9A5
-1.942	1.06E-02	SLTM
-2.03	9.20E-03	SMAP1
-1.706	3.74E-02	SMC2
-1.903	1.93E-02	SMC4
-1.7	4.00E-02	SMC5
-2.001	6.75E-03	SMCHD1
2.11	9.95E-03	SMPD1
2.92	1.00E-03	SNCG

2.951	2.65E-02	SNORA48
1.573	1.16E-02	SNORD21
3.485	4.28E-02	SNORD36A
-2.245	2.95E-02	SNORD97
2.449	2.86E-02	SNPH
-1.731	4.13E-02	SNRPF
-1.762	3.03E-02	SOGA1
1.926	4.43E-02	SOX8
-3.428	4.61E-02	SPATA17
-1.914	3.25E-02	SPC25
2.786	1.12E-02	SPON1
-1.729	3.91E-02	SPTSSA
-1.842	2.20E-02	SRF
-2.006	7.80E-03	SRRT
-1.742	3.16E-02	SRSF1
-2.006	1.20E-02	SRSF3
-1.99	6.45E-03	SRSF6
-1.742	2.88E-02	SRSF7
-1.698	3.62E-02	STAG1
-1.907	3.40E-02	STC1
-1.938	1.24E-02	STIL
1.768	4.96E-02	STK39
2.386	2.61E-02	STX1B
2.243	1.77E-02	SULT1A1
-1.735	4.74E-02	SUPT16H
-1.822	3.52E-02	SUV39H1
-1.951	1.69E-02	SYNCRIP
2.61	1.29E-02	SYNGR3
-3.673	1.45E-03	SYT1
-2.005	9.40E-03	TAF9B
-1.693	4.11E-02	TARDBP
2.088	2.03E-02	TBC1D10A
1.878	2.51E-02	TBC1D17
-7.789	4.45E-02	TCAP
-1.972	8.15E-03	TCF7
4.87	3.65E-02	TEX22
-2.462	1.47E-02	TEX30
-1.688	4.23E-02	TFAM
-1.979	1.28E-02	TFRC
-1.97	3.58E-02	TGS1
2.091	3.65E-02	THSD4

-1.924	1.30E-02	THUMPD1
-2.435	1.43E-02	TIPIN
-3.275	5.00E-05	TM4SF18
1.969	3.14E-02	TMBIM4
2.534	6.35E-03	TMC6
1.907	2.32E-02	TMEM129
1.835	2.93E-02	TMEM132A
-3.796	2.27E-02	TMEM145
1.935	2.19E-02	TMEM158
4.862	4.13E-02	TMEM221
2.732	9.00E-04	TMEM59L
-2.102	6.05E-03	TMOD3
-3.27	5.00E-05	TMX3
-5.135	3.05E-03	TNFAIP3
-3.362	5.00E-05	TNFRSF10D
-6.887	3.92E-02	TNNC1
-1.938	9.60E-03	TNPO1
-1.7	4.13E-02	TNRC6A
-1.674	4.63E-02	TOMM34
1.819	4.32E-02	TP53I13
2.259	1.87E-02	TPBG
-3.147	2.36E-02	TPI1P2
-1.847	4.90E-02	TPM1
-2.033	7.60E-03	TRA2A
-2.676	1.00E-04	TRA2B
1.896	4.96E-02	TRIM16
-2.009	1.21E-02	TRIM55
-1.665	4.98E-02	TRIP13
-1.863	3.52E-02	TRMT10C
-1.968	1.95E-02	TRMT6
2.068	4.03E-02	TRPV2
2.072	4.69E-02	TUBB3
-1.768	3.29E-02	TWISTNB
-1.747	3.19E-02	U2AF1
-1.832	1.62E-02	UAP1
1.783	3.35E-02	UAP1L1
-1.765	2.84E-02	UBE2C
-2.225	1.50E-02	UBE2D4
-1.679	4.96E-02	UBE2V2
-2.165	2.70E-03	UHRF1
2.137	4.59E-02	ULBP2

-1.793	2.50E-02	UPF2
-2.357	2.00E-03	UPF3B
2.74	2.70E-02	UPK3B
-1.864	1.53E-02	USP1
-2.027	1.06E-02	USP22
-1.867	2.71E-02	USP37
-1.864	3.14E-02	VGLL4
-1.782	4.51E-02	VRK1
-1.656	4.53E-02	WAPAL
-1.796	2.91E-02	WDHD1
-1.724	3.51E-02	WDR43
-2.433	2.35E-03	WDR76
2.309	4.70E-03	WIPI1
-2.872	8.50E-04	XRCC2
1.868	4.71E-02	YPEL2
1.881	3.89E-02	YPEL3
2.579	3.89E-02	ZBTB42
2.148	7.10E-03	ZBTB7B
-1.727	4.42E-02	ZC3H7A
-1.702	4.95E-02	ZC3HAV1L
2.52	4.61E-02	ZHX2
-1.837	1.74E-02	ZMIZ2
-2.027	1.15E-02	ZNF107
-2.937	9.75E-03	ZNF235
-2.189	7.25E-03	ZNF267
-2.008	1.42E-02	ZNF326
-1.867	3.16E-02	ZNF367
-1.896	1.46E-02	ZNF395
2.126	2.48E-02	ZNF446
2.405	1.09E-02	ZNF467
-1.964	1.95E-02	ZNF480
3.443	5.55E-03	ZNF516
-2.013	4.13E-02	ZNF551
2.176	1.42E-02	ZNF581
2.797	1.98E-02	ZNF641
-2.75	3.05E-03	ZNF724P
-2.235	3.84E-02	ZSCAN12
1.785	4.93E-02	ZSWIM4
-2.205	4.10E-03	ZWINT

Supplementary Table 2: Network ID assigned on the basis of network score, genes involved in the network, score of the network, focus molecules representing the number of genes in our dataset that are present and top diseases and functions associated with the genes.

ID	Molecules in Network	Score	Focus Molecules	Top Diseases and Functions
**1**	ALYREF,BARD1,BCLAF1, CCAR1,CHD3,CUL7,DDX39A,ERCC6L,HNRNPH3,HNRNPU,KCNK3,KIFC2,LOC102724594/U2AF1,MALAT1, NCBP1,NOL9,NOL11,NSRP1,PCF11,Pkc(s),PNN,RBMX, SAFB,SAFB2,SMCHD1,SRRT,SRSF1,SRSF3,SRSF6,SRSF7,STC1,TRA2A,TRA2B,UPF3B,WDR43	54	34	RNA Post-Transcriptional Modification, Molecular Transport, RNA Trafficking
**2**	AK4,AP3M1,ARFGEF3,CDC7,CKAP2,CMSS1,DCLRE1B,EGFR,EIF3M,GINS1,GINS2,GINS3,GINS4,HES4,HMGB3,KCTD9,Mcm,MCM4,MCM5,MCM10,MCMBP,MT1M,NT5DC2,nucleotidyltransferase,OAS2,ORC6,PLD3,POLA1,POLD3,PRIM1,PRIM2, Primase,PRMT3,TRMT10C, WDHD1	49	32	Cellular Assembly and Organization, Cancer, Hematological Disease
**3**	ARSA,ATM/ATR,BLVRB,BRE,Bvr,CLIP2,CLSPN,CRLF1,EXO5,FAIM,GJC1,KCNJ4, KIAA1524,LIN7C,LUC7L3, MIB2,MRN,NFkB (complex),PDCD5,PIM3,PLAG1,PPIC,PPM1D,PPME1,PSPC1,RAB31,Rfc,RFWD3,RIF1,RIPK4,RPA,SLC22A18,SOGA1,TIPIN,TMX3	41	29	Cell Cycle, DNA Replication, Recombination, and Repair, Neurological Disease
**4**	AEN,ALOXE3,ANKRD32,ATP13A2,BCR (complex),BRINP3,CACNA2D2,CKAP2L,CXCL5,ESCO2,	41	29	Cancer, Connective Tissue Disorders, Developmental Disorder

	FAM111B,FAM173B,GADD45A,Iga,Immunoglobulin,KIF18A,KIF1BP,LENG8,MAP1LC3,MHC Class II (complex),MTFR2,NFIB,NRROS,NUPR1,POLE2,PRND,PVT1,RIMKLA,SLC6A6,SRC (family),SUV39H1,SYNCRIP,TFRC,UAP1,ZNF581			
**5**	ASNS,CDC6,Cdc25B/C,CDK1,CDK2,FUS,GEN1,HIST2H2BF,HNRNPM,IGSF8,Interferon alpha,KCNAB2,KIF23,LLPH,LYAR,MCM3,MYOF,NAT14,NPDC1,p85 (pik3r),PCNA,PLK1,PRPF3,PRPF4,PRPF38B,PRPF4B,PSMD12,RABAC1,Rb,RNA polymerase II,RPS21,SEC24D,SF3B4,SFPQ,snRNP	41	29	RNA Post-Transcriptional Modification, Cellular Assembly and Organization, Cell Cycle
**6**	ANKRD26,ANKRD28,ATAD2,ATAD5,ATPase,BLM,BRCA1,BRCC3,BTF3,Cbp/p300,DHX9,DNA2,DUB,ERO1LB,EXO1,FEN1,FIGNL1,G3BP1,Gsk3,GTF2A1,Holo RNA polymerase II,I kappa b kinase,KIF20B,LMNTD1,MCM2,NMI,PA2G4,PGK1,RAB22A,RAD51,SUPT16H,TAF9B,TFIIE,USP1,USP22	39	28	DNA Replication, Recombination, and Repair, Cell Cycle, Cancer
**7**	ABLIM1,Angiotensin II receptor type 1,ANLN,APLP1,Arf,BARX2,CBFB,CDCA7,CDKN1A,CEP55,Cofilin,CTNNAL1,DROSHA,Erm,ETS1,F Actin,HIGD1A,ICAM5,IKZF2,LGALS3BP,LPCAT3,MTERF1,Notch,OLFM2,P4HA1,Pias,PITPNM1,Rock,Secretase gamma,Smad,SPON1,SRF,TCF7,TMBIM4,TPM1	33	25	Cell-mediated Immune Response, Cellular Development, Cellular Function and Maintenance

**8**	AASDHPPT,DNASE2,GRPEL1,HLA-A,HLA-B,HLA-C,IFIT1,IFIT3,Ifn,IFN alpha/beta,IFN Beta,IFN type 1,Ifnar,IL-2R,IL12 (complex),JAK,KDM6B,LDHA,MHC Class I (complex),MHC I-Î±,NAA15,NT5C2,NUDT18,OASL,PAICS,PHGDH,POLR3G,PRRG4,SDPR,SEZ6L2,SHC1,SNORD21,SNRPF,Tlr,UPF2	31	24	Dermatological Diseases and Conditions, Immunological Disease, Inflammatory Disease
**9**	AMOTL2,BTG2,CDCA5,CDK2-CyclinE,ERBB,ERK,ETS,FRS3,GRB7,HOXB9,IGFBP6,KIF4A,KLF9,LIN9,MAGOHB,MKNK2,MYBL1,N-cor,NCAPG,NCAPG2,NCAPH,NRG (family),PITX1,PLIN2,Rxr,SERCA,SH3RF2,SMC2,SMC4,STAG1,T3-TR-RXR,thyroid hormone receptor,TMOD3,VitaminD3-VDR-RXR,WAPAL	30	24	DNA Replication, Recombination, and Repair, Cell Cycle, Cellular Assembly and Organization
**10**	C15orf48,CEBPB,CFD,Cg,CHRAC1,DNAJA1,EPAS1,EZH2,FAM198B,GTPase,HIF1A,Histone h3,HOXB8,Hsp70,Hsp90,HSPA5,HSPH1,Ige,IL12 (family),MANF,NIFK,NR2F2,PDIA5,Proinsulin,RAPGEF6,RCBTB2,RFC3,SMAP1,SNCG,STAT5a/b,TFAM,TM4SF18,TMEM132A,VRK1,ZNF267	30	26	Cellular Compromise, Embryonic Development, Organ Development
**11**	ARHGAP32,ARRDC3,CCRN4L,CHAF1B,CHST3,CHST11,CLNS1A,CLOCK,Cyclin D1/cdk4,DBP,EED,EIF4EBP2,EPC1,Fcer1,GATA2,Gm-csf,Hat,HISTONE,histone deacetylase,Histone	29	24	Behavior, Carbohydrate Metabolism, Nervous System Development and Function

	h4,KAT6B,Mapk,MORF4L1,MutS alpha,NPAT,PER2,PIGA,PIGQ,SHC2,SLC20A1,sulfotransferase,SULT1A1,TGS1,TIP60,ZBTB7B			
**12**	AATF,ADCY5,APC (complex),BNIP3L,Cdc25,CDC25A,Cdk,CHEK1,CKS1B,COPS3,Cyclin B,DTL,E3 RING,FANCM,FBXL16,FBXO5,FBXW4,GNAO1,Lh,LIPA,LMO7,Lpa receptor,Pka,PTP4A1,PTPase,PTPRU,RAB32,RASAL2,RBL1,SKP1,SNPH,Sod,TNRC6A,tyrosine kinase,UBE2C	29	24	Cell Cycle, DNA Replication, Recombination, and Repair, Connective Tissue Development and Function
**13**	Akt,ANKRD1,atypical protein kinase C,CDK4/6,CLIP4,Collagen type VI,CREB3L3,DES,INPP4B,JUN/JUNB/JUND,LPXN,MICAL1,MYPN,N-Cadherin,Neurotrophin,NFkB (family),NRP1,PBXIP1,PLAC8,PLEKHA2,Plexin A,PLXNA3,PVRL2,RIN2,RRAS,Sema3,SEMA3A,SEMA3C,SEMA3D,SLC22A17,SLC39A10,TCAP,TRIM55,UBE2,Ube3	29	23	Cell-To-Cell Signaling and Interaction, Nervous System Development and Function, Cell Morphology
**14**	ADAMTS4,Alpha catenin,Ap1,ARHGAP31,ARRDC2,BCAT1,BIRC3,CaMKII,CASP2,DEPDC1,FURIN,Ikb,IKK (complex),Ikk (family),LMNB1,MAP2,NFAT (complex),NOP58,PP1-C,Ppp2c,PPP4R1,PRKAC,PROCR,QPCTL,Rac,RBCK1,RIPK1,RNF138,S100A6,SMC5,TARDBP,TCR,TNFAIP3,UBE2D4,Ubiquitin	27	22	Gastrointestinal Disease, Hepatic System Disease, Inflammatory Disease
**15**	aldehyde dehydrogenase (NAD),ALDH,ALDH1A1,AL	25	22	Developmental Disorder, Neurological Disease, Organismal

	DH1A3,ALDH3B1,ALDH9A1,BIRC7,c-Src,CCNF,CHRD,Ctbp,DDX11,DKK1,DVL1,Ecm,Frizzled,Gli,HECW1,Hedgehog,HELLS,Integrin alpha 3 beta 1,KIF11,KREMEN2,LRP,LRP10,LTBP4,MSI1,PPP6R3,PRICKLE1,PTCH1,STIL,TCF,TMEM158,Vegf,Wnt			Injury and Abnormalities
**16**	26s Proteasome,Actin,AGGF1,Alpha Actinin,ANXA10,BAG3,BEAN1,BMS1,caspase,CD3,chemokine,Ck2,cytochrome C,DPCD,GTF3C6,HMGB1,Ifn gamma,MACROD1,MAN2B1,MAPK12,MDM2,MT1A,NAP1L4,NPIPA8 (includes others),NXF1,OGDHL,ORAI3,PNPLA6,PPP1R26,PRPS2,RAG1,RAG2,SP100,TPBG,ZNF326	24	22	Developmental Disorder, Connective Tissue Disorders, DNA Replication, Recombination, and Repair
**17**	ARID1A,BMF,Cdc2,CDK1/2,Collagen Alpha1,Collagen type V,Cyclin A,Cyclin A/Cdk2,Cyclin E,DARS,DDR2,E2f,E2F8,EXOSC2,Fetal Hemoglobin,GC-GCR dimer,GSPT1,Il3r,Jnk,KNTC1,LTBP1,MELK,MRC2,MSLN,NET1,PMEPA1,SESN2,SKA2,SKA3,Smad2/3,Stat3-Stat3,SWI-SNF,TNFRSF10D,USP37,WDR76	23	20	Cell Cycle, Hereditary Disorder, Neurological Disease
**18**	ABCC4,Alpha tubulin,Beta Tubulin,CACNB3,Caveolin,CENPE,Cytokeratin,DNMBP,Dynamin,Dynein,FAM162A,GABARAPL1,HK2,HLA Class I,KIR,KRT13,KRT16,MALL,	23	20	Hereditary Disorder, Neurological Disease, Cell Death and Survival

	PCNT,PDK1,Pgk,PI3K (complex),RPS6KA,SCN1B, SPTSSA,STX1B,Syntaxin,SYT1,TBC1D17,TBC1D10A,TUBB3,tubulin (complex),tubulin (family),ULBP2,Vdac			
**19**	ADI1,AGRN,BCAR1,BRCA2,BRCA1-BRCA2-FANCD2-FANCN-RAD51,COL18A1,Collagen type I,Collagen type IV,Collagen(s),CRELD2,CTSL,DDX41,elastase,FARP2,Filamin,FYN,GDF15,GPIIB-IIIA,HCN2,HERPUD1,Integrin,Integrin alpha 4 beta 1,IntegrinÎ±,ITGB4,Laminin, Laminin1,MMP14,Na+,K+ -ATPase,NCOA5,ORC1,PARVA,Ptk,RAD51C,Rap1,XRCC2	23	20	Cellular Movement, Cell Cycle, DNA Replication, Recombination, and Repair
**20**	20s proteasome,Calcineurin A,Calcineurin protein(s),CHORDC1,CITED2,DLL1,DNA-methyltransferase,FGF5,FHL1,FOSL1,Hdac,HDAC5,HDAC9,HEATR6,HSP,LHX1,MARCKSL1,MB,MEF2,MHC CLASS I (family),MNT,MYH7,Myosin,Nfat (family),NRIP1,ODC1,OSR1, PDK4,PPIF,PPP1R2,Ras,Rsk, SAT1,TOMM34,Tropomyosin	22	22	Molecular Transport, Small Molecule Biochemistry, Cardiovascular System Development and Function
**21**	Alp,AMPK,CAV1,CPSF1,Creb,CXCL2,EGR1,EGR3,EHF,Fgf,Fgfr,GBX2,HMOX1,INSIG1,Insulin,ITGA5,KLF4,MAP2K1/2,MASTL,MCU,Mek, MTORC1,MYB,Nos,NOS1AP,p70 S6k,Pdgf (complex),PDGF BB,PLC gamma,PP1 protein complex	22	19	Cell Death and Survival, Cell-mediated Immune Response, Cellular Development

	group,RPS6KA2,SERPINA3,SHH,Tgf beta,TRPV2			
**22**	AMER1,AMMECR1,ANKS1A,AUNIP,C19orf26,CDK6,CLTC,DLG4,EGR1,ELAVL1,EPO,FAM13B,FBXW11,FEM1C,GATA3,GSK3B,GSPT1,KDR,KITLG,KLHL17,KLHL24,MESDC1,MNT,N4BP2L1,NRAS,PAX6,PIM1,PRKCD,RBX1,REXO1,RPP14,TWISTNB,YTHDF1,ZNF395,ZSWIM4	22	19	Cell Cycle, Cellular Development, Hematological System Development and Function
**23**	AKAP12,Collagen type II,CYBA,Cyclin D,ERK1/2,GPC1,Hla-abc,HMMR,Igf,NADPH oxidase,NCK,NDC80,NOG,Pak,PCSK1N,PDGF (family),Pdgf Ab,PDGF-AA,PDGFB,PDGFC,PFKFB4,PHAX,PIK3R3,Pkg,RGS5,RTN4R,SLC9A5,SMAD1/5,Smad1/5/8,Sos,SPC25,VAV,Vla-4,ZHX2,ZWINT	20	18	Cell Cycle, Cellular Assembly and Organization, DNA Replication, Recombination, and Repair
**24**	ABCG1,ABTB1,AChR,ADM,ALDOC,CD9,CTSC,Fc gamma receptor,Fc receptor,FCGRT,FGFR1OP,glutathione peroxidase,GOT,hemoglobin,IgG,IgG1,Igg3,IgG2a,Igh (family),Igm,Ldh (complex),MHC,MHC II,MIR124,OSBPL3,PI3K (family),POLQ,PPP1R15A,RELA,SFN,SHCBP1,SLC2A1,SLC30A1,TMC6,TRIM16	20	18	Tissue Morphology, Embryonic Development, Lymphoid Tissue Structure and Development
**25**	alcohol group acceptor phosphotransferase,APC/APC2,Arp2/3,BBC3,CAK,calpain,CDA,CDK6,CDK1-Cyclin B,Collagen type III,GADD45,Gcn5l,HIST1H1B,HIST1H1E,Histone H1,ID4,Importin	18	17	Cardiovascular System Development and Function, Cell Morphology, Hematological System Development and Function

	alpha,Importin beta,JMY,KIF1A,Nucleoporin,NUP50,NUP153,NUP155,P-TEFb,P38 MAPK,PIM1,PP2A,STK39,Tap,TNNC1,TNPO1,Transportin,UHRF1,VGLL4			

Supplementary Table 3: Ten enriched canonical pathways; ratio of the genes present in our dataset to the number of genes that map to that pathway in the IPA library, z-score and p-values. A positive z-score implies activation of the pathway while a negative score implies predicted inhibition; NaN stands for "Not a Number" where the activation state could not be inferred. Genes presented in red, also identified by the ↑ symbol, indicate upregulation of the genes in the dataset, genes represented in black are downregulated.

	Canonical pathway	Genes	Ratio	Z score	P value
1.	Chromosomal Replication Pathway	CDC6, CDC7, CDK2, CDK6, MCM2, MCM3, MCM4, MCM5, ORC1, ORC6	10/27 (0.37)	NaN	1.66E-08
2.	DNA Damage Signaling (BRCA1-mediated)	ARID1A, BARD1, BLM1, BRCA1, BRCA2, BRCC3, BRE, CDKN1A, CHEK1, FANCM, GADD45A, PLK1, RAD51, RBL1, RFC3	15/78 (0.192)	-2.53	1.01E-07
3.	G2/M DNA Damage Regulatory Pathway	BRCA1, CDK1, CDKN1A, CHEK1, CKS1B, GADD45A, MDM2, PLK1, PPM1D, SFN, SKP1	11/49 (0.224)	0.302	1.01E-06
4.	ATM Signaling	BLM, BRCA1, CDC25A, CDK1, CDK2, CDKN1A, CHEK1, GADD45A, MDM2, RA51, SMC2	11/59 (0.186)	-1.897	7.02E-06
5.	P53 Signaling	↑BBC3, BRCA1, CDK2, CDKN1A, CHEK1, GADD45A, HDAC9, HIF1A, ↑JMY, MDM2, PCNA, PIK3R3, SFN, STAG1	14/98 (0.143)	0.632	1.07E-05
6.	G1/S Cell Cycle Regulatory Pathway	CDC25A, CDK2, CDK6, CDKN1A, ↑HDAC5, HDAC9, MDM2, PA2G4, RBL1, SKP1, SUV39H1	11/64 (0.172)	0.333	1.58E-05
7.	Homologous Recombination Repair Pathway	BRCA1, BRCA2, GEN1, POLA1, RAD51	5/14 (0.357)	NaN	9.08E-05
8.	Pancreatic Adenocarcinoma Signaling	BRCA2, CDK2, CDKN1A, EGFR,	13/106 (0.123)	-0.333	1.11E-04

		↑HMOX, MDM2, PA2G4, PDGFC, PIK3R3, ↑PLD3, RAD51, RELA			
9.	Mismatch Repair Pathway	EXO1, FEN1, PCNA, RC3	4/16 (0.25)	NaN	2.14E-03
10	Hif1α Signaling	HIF1A, LDHA, MDM2, ↑MMP14, PDGFC, PIK3K3, ↑RRAS, SLC2A1	8/102 (0.078)	NaN	3.08E-02

Supplementary Table 4: Table of primers used in our study to validate the RNA-seq data along with the primers used for ChIP assay.

S.No.	Gene	Primer Sequence
1.	GAPDHF	CAC CGT CAA GGC TGA GAA C
	GAPDHR	GAC TCC ACG ACG TAC TCA G
2.	ADMF	ATG AAG CTG GTT TCC GTC G
	ADMR	GAC ATC CGC AGT TCC CTC TT
3.	SHHF	CTC GCT GCT GGT ATG CTC G
	SHHR	ATC GCT CGG AGT TTC TGG AGA
4.	MDM2F	GAA TCA TCG GAC TCA GGT ACA TC
	MDM2R	TCT GTC TCA CTA ATT GCT CTC CT
5.	ALDH1A1F	CTG CTG GCG ACA ATG GAG T
	ALDH1A1R	CGC AAT GTT TTG ATG CAG CCT
6.	HDAC5F	TCT TGT CGA AGT CAA AGG AGC
	HDAC5R	GAG GGG AAC TCT GGT CCA AAG
7.	BBC3F	GCC AGA TTT GTG AGA CAA GAG G
	BBC3R	CAG GCA CCT AAT TGG GCT C
8.	MYBF	TCA GGA AAC TTC TTC TGC TCA CA
	MYBR	AGG TTC CCA GGT ACT GCT
9.	LDHAF	ATG GCA ACT CTA AAG GAT CAG C
	LDHAR	CCA ACC CCA ACA ACT GTA ATC T
10.	HIF1AF	GAA CGT CGA AAA GAA AAG TCT CG
	HIF1AR	CCT TAT CAA GAT GCG AAC TCA CA
11.	KLF4F	CAG CTT CAC CTA TCC GAT CCG
	KLF4R	GAC TCC CTG CCA TAG AGG AGG
12.	GLUT1F	AAC TCT TCA GCC AGG GTC CAC
	GLUT1R	CAC AGT GAA GAT GAT GAA GAC
13.	EGFRF	CCC ACT CAT GCT CTA CAA CCC
	EGFRR	TCG CAC TTC TTA CAC TTG CGG
14.	RELAF	GTG GGG ACT ACG ACC TGA ATG
	RELAR	GGG GCA CGA TTG TCA AAG ATG
15.	RELMYBPF2	GGC TTT GCT CCA GAC CCG GC
	RELMYBPR2	GGT GGG TCC GCC GAT TAC TCA C
16.	EGFRMYBPF	GTC CAG AGG GGC AGT GCT G
	EGFRMYBPR3	GAC CTA GTC TCC GGC GGG
